# Quantitative analysis of inhibitor‐induced assembly disruption in human UDP‐GlcNAc 2‐epimerase using mass photometry

**DOI:** 10.1002/pro.70335

**Published:** 2025-10-16

**Authors:** Nico Boback, Jacob Gorenflos López, Christian P. R. Hackenberger, Santiago Di Lella, Daniel C. Lauster

**Affiliations:** ^1^ Freie Universität Berlin Institute of Pharmacy, Biopharmaceuticals Berlin Germany; ^2^ Leibniz‐Forschungsinstitut für Molekulare Pharmakologie (FMP) Berlin Germany; ^3^ Humboldt‐Universität zu Berlin Department of Chemistry Berlin Germany; ^4^ Universidad de Buenos Aires—Consejo Nacional de Investigaciones Científicas y Técnicas Instituto de Química Biológica de la Facultad de Ciencias Exactas y Naturales Ciudad de Buenos Aires Argentina

**Keywords:** assembly inhibition, mass photometry, molecular docking, protein assembly, sialic acid biosynthesis, UDP‐GlcNAc 2‐epimerase

## Abstract

Uridine diphosphate *N*‐acetylglucosamine (UDP‐GlcNAc) 2‐epimerase (GNE)/*N*‐acetylmannosamine kinase is the rate‐limiting enzyme in sialic acid biosynthesis and a promising therapeutic target. We applied mass photometry (MP) to investigate GNE oligomerization and its modulation by three small‐molecule inhibitors (C5, C13, and C15). Substrate‐binding (UDP‐GlcNAc) stabilized tetramer formation by increasing dimer–dimer affinity 98‐fold. All inhibitors destabilized tetramers in a concentration‐dependent manner, with IC_50_ values in the low micromolar range. Using a modified Cheng–Prusoff equation, IC_50_ values were converted into *K*_B,app_ values. Schild analysis and Operational Model of Allosterically Modulated Agonism were applied to estimate an apparent *K*_B,app_ value and assess cooperative inhibition effects. Molecular docking confirmed competitive binding for all inhibitors and helped rationalize observed potency trends. While MP has previously been used to study protein assembly, our work demonstrates its applicability for the label‐free, quantitative characterization of small‐molecule inhibitors affecting protein oligomerization. These findings provide a foundation for further mechanistic studies and underscore the potential of MP in drug‐target interaction profiling.

## 1 INTRODUCTION

The *de novo* synthesis of sialic acid (SA), a key sugar involved in cellular processes such as signaling, adhesion, and immunity (Irons et al., 2024; Schauer, 2009; Stencel‐Baerenwald et al., 2014), is governed by the bifunctional enzyme uridine diphosphate‐*N*‐acetylglucosamine 2‐epimerase/*N*‐acetylmannosamine kinase (GNE/MNK). As the rate‐limiting enzyme in SA biosynthesis, GNE/MNK converts uridine diphosphate *N*‐acetylglucosamine (UDP‐GlcNAc), a product of the hexosamine biosynthetic pathway, into *N*‐acetylmannosamine (ManNAc), which is subsequently phosphorylated under adenosine triphosphate consumption to form ManNAc‐6‐phosphate (ManNAc‐P_6_). Two additional conversion steps, catalyzed by sialic acid synthase (SAS) and sialic acid phosphatase (SAP), yield the final product, Neu5Ac, which can be further converted by CMP‐Neu5Ac synthetase into the feedback inhibitor CMP‐Neu5Ac (Chen et al., 2016; Gorenflos López, Schmieder, et al., 2023; Hinderlich et al., 2015). Individual GNE subunits primarily form enzymatically inactive homodimers, but the presence of UDP‐GlcNAc promotes the formation of an active homotetrameric state (Chen et al., 2016; Gorenflos López, Dornan, et al., 2023; Hinderlich et al., 2015). However, even in its active form, GNE exhibits low catalytic efficiency (*k*_cat_ = 11.8 ± 2.0 s^−1^, and *K*_M_ = 33.1 ± 4.2 μM). In contrast to the substrate, the feedback inhibitor CMP‐Neu5Ac stabilizes an inactive tetrameric conformation by binding at the dimer–dimer interface, thereby downregulating enzyme activity (Chen et al., 2016). This assembly‐state‐dependent regulation of GNE is a central focus of the present study.

**FIGURE 1:**
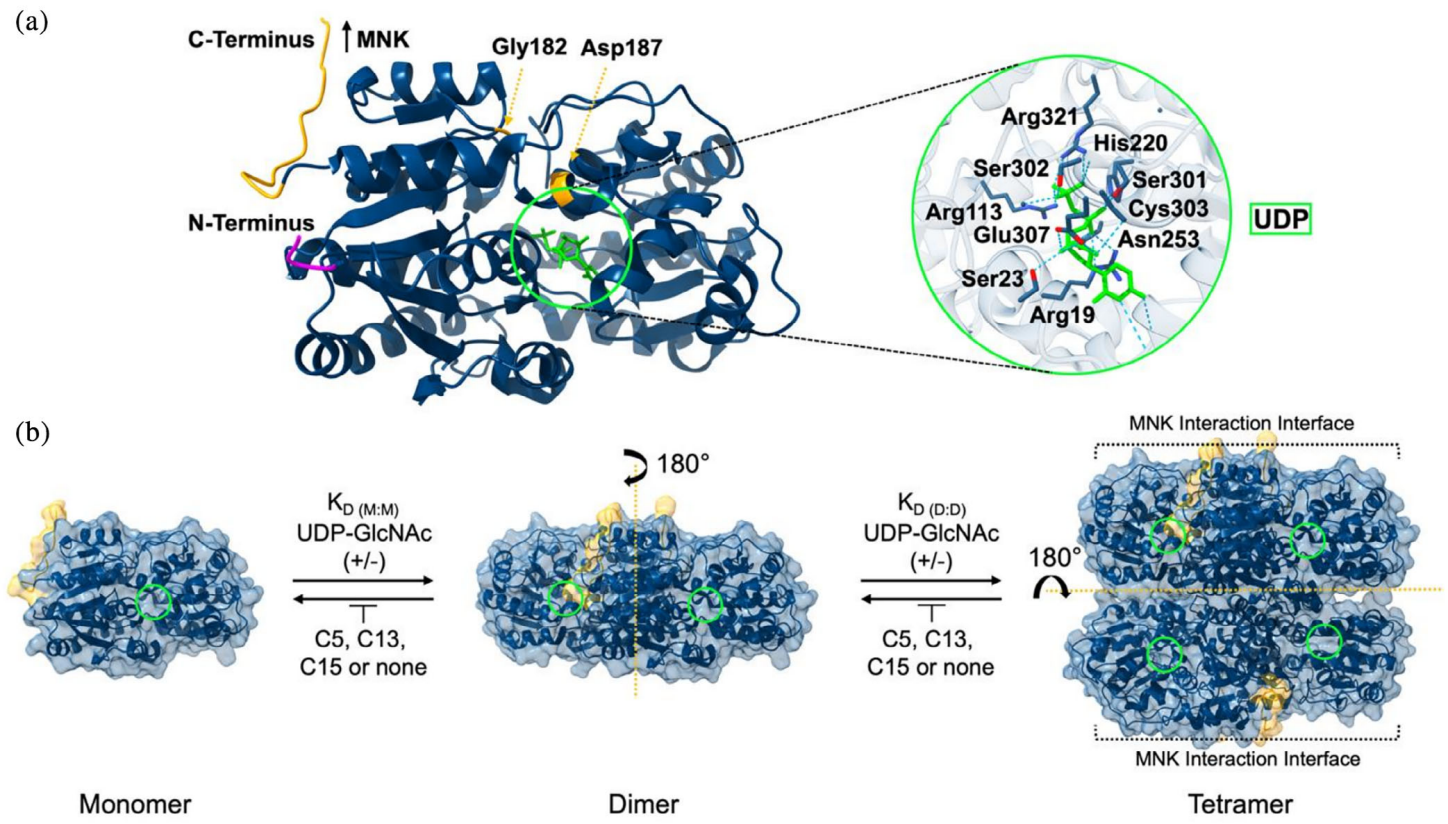
Structural insights into uridine diphosphate‐*N*‐acetylglucosamine 2‐epimerase (GNE)‐substrate binding and tetramer assembly dynamics in the presence of substrate or inhibitors. (a) Crystal structure of monomeric GNE bound to UDP (green), highlighting key protein residues involved in substrate interaction. (b) GNE assembly equilibria between monomer–dimer (*K*_D(M:M)_), and dimer–tetramer (*K*_D(D:D)_) states, characterized by distinct dissociation constants in the presence or absence of uridine diphosphate‐*N*‐acetylglucosamine (UDP‐GlcNAc) and inhibitors (C5, C13, and C15). The substrate‐binding site is indicated by a green circle. The *C*‐terminal region connecting GNE and *N*‐acetylmannosamine kinase (MNK) subunits in the full‐length protein and key residues increasing linker flexibility are highlighted in yellow. The *N*‐terminal region is marked in magenta. The monomeric structure and protein assemblies of GNE are derived from the protein data bank (Protein data bank (PDB, ID: 4zht) (Chen et al., 2016). Structural visualizations were generated using UCSF (Pettersen et al., 2021) ChimeraX‐1.9.

The crystal structure of GNE, derived from a complex with uridine diphosphate (UDP, substrate component) and CMP‐Neu5Ac (feedback inhibitor), reveals that each GNE monomer (~43 kDa) contains a substrate‐binding site within its core. UDP interacts at this site through multiple salt bridges and hydrogen bonds (Figure 1a). GNE forms dimers through non‐covalent interactions involving hydrophobic side chains from helices ɑ3, ɑ4, and ɑ5 at the *N*‐terminal region (Figure 1a, magenta). These dimers further associate into tetramers via a 2 + 2 binding configuration (Figure 1b), stabilized by a hydrophobic core at helix ɑ3 near the tetramer interface and multiple salt bridges (Chen et al., 2016). The MNK subunit connects to the *C*‐terminus of the GNE subunit (Figure 1a, yellow), establishing the full‐length protein “Master Regulator,” forming homodimers in the absence or homotetramers in the presence of substrate or feedback inhibitor (Chen et al., 2016; Hinderlich et al., 2015; Kurochkina et al., 2009). The GNE subunits *C*‐terminal connecting region exhibits high flexibility, facilitated by a hinge region involving Gly182 and Asp187 (Kurochkina et al., 2009). A full‐length structural model suggests that the active sites of GNE and MNK are positioned in close proximity, potentially forming an internal epimerase‐kinase channel to enable efficient and protected transfer of intermediates (Chen et al., 2016; Hinderlich et al., 2015).

Targeting the GNE/MNK enzyme—either as a whole or through its individual protein subunits—offers a potential strategy for therapeutic intervention. This approach has been demonstrated in the modulation of SA densities on cell surfaces (Willems et al., 2019; Wratil et al., 2014), impacting cellular signaling (Irons et al., 2024; Schauer, 2009), and host‐cell susceptibility to pathogens, including influenza A virus (Schauer, 2009; Stencel‐Baerenwald et al., 2014). In the context of influenza A virus inhibition, reducing SA densities on the respiratory epithelium has proven effective. Strategies such as CRISPR‐based GNE knockouts (Ma et al., 2023) and sialidase fusion proteins (Belser et al., 2007) have successfully inhibited viral infection. However, unlike permanent gene knockouts, small‐molecule inhibitors of GNE could provide a reversible and controlled alternative for modulating SA biosynthesis, offering greater therapeutic flexibility.

Previous enzyme activity assays from our group established that GNE's quaternary structure directly influences its enzymatic activity. UDP‐GlcNAc binding promotes tetramer formation, while small‐molecule inhibitors disrupt oligomerization by interfering with substrate binding (Gorenflos López, Dornan, et al., 2023). Additionally, the feedback inhibitor CMP‐Neu5Ac was shown to stabilize the tetrameric, inactive state via allosteric modulation, altering UDP‐GlcNAc affinity at the substrate‐binding pocket (Chen et al., 2016).

In our recent high‐throughput screening of 68,640 small‐molecule compounds (Gorenflos López, Dornan, et al., 2023), we identified three potent, non‐carbohydrate‐based UDP‐GlcNAc 2‐epimerase inhibitors: C5, C13, and C15. These inhibitors exhibited high potency and cell culture medium stability, with C5 derived from the purine base xanthine scaffold, while C13 and C15 share a pyrimidinone core, structurally similar to UDP‐GlcNAc, which is linked via a vinyl to an aromatic system. Supported by different detection techniques (e.g., mass photometry [MP] and hydrogen‐deuterium exchange mass spectrometry [HDX‐MS]), we showed further that these inhibitors reveal distinct destabilizing effects on GNE assembly (Gorenflos López, Dornan, et al., 2023). HDX‐MS results suggested that all three inhibitors compete with UDP‐GlcNAc for the substrate‐binding site, leading to protein disassembly. However, C13 and C15 also induced unique conformational changes, distinct from those caused by C5 or the substrate, enhancing negative cooperativity in protein assembly and thus inhibitor potency. Qualitative analysis confirmed a progressive increase in inhibitory potency from C5 to C13 to C15. Despite these insights, the precise molecular mechanisms underlying GNE assembly inhibition remain only partially understood, as different inhibitors may disrupt oligomerization through diverse pathways (Gorenflos López, Dornan, et al., 2023).

To further explore the effects of these inhibitors, we previously employed MP to study the stability of GNE tetramers and dimers at a single inhibitor concentration (10 μM) in the presence or absence of UDP‐GlcNAc (10 μM) (Gorenflos López, Dornan, et al., 2023). This technique detects mass‐dependent signals from individual proteins or protein assemblies by measuring interferometric light scattering at a glass‐solution interface (Fineberg et al., 2020; Young et al., 2018). Our findings demonstrated that C5, C13, and C15 all disrupted tetrameric GNE, while C13 and C15 also destabilized dimeric assemblies, a property not observed for C5. Consistent with enzyme activity assays, C13 and C15 exhibited higher potency than C5, suggesting a stronger influence on GNE disassembly (Gorenflos López, Dornan, et al., 2023). However, the underlying differences in potency and inhibition mechanisms remain incompletely understood.

Building upon our initial findings, the present study was designed to systematically characterize the inhibition mechanisms of C5, C13, and C15 using MP. Although this technique has been applied to various biological systems, including bovine serum albumin (BSA) (Young et al., 2018), plant‐derived 2‐cysteine peroxiredoxins (Liebthal et al., 2021), transmembrane transport channels (KcsA and OmpF), membrane oxidoreductases (Olerinyova et al., 2021), antibodies (IgG) (den Boer et al., 2022; Soltermann et al., 2020), SARS‐CoV‐2 spike proteins (Burnap & Struwe, 2022), and surfactant proteins A and D (Avcibas et al., 2024), studies examining the inhibition of protein assembly using MP remain limited (Dimitrijevs et al., 2023; Gorenflos López, Dornan, et al., 2023). Compared to conventional methods such as gel electrophoresis (Gorenflos López, Schmieder, et al., 2023), analytic size‐exclusion chromatography (aSEC), and HDX‐MS (Gorenflos López, Dornan, et al., 2023), MP offers key advantages, including low sample consumption, label‐free single‐molecule detection, and a broad molecular mass range (40 kDa to ~10 MDa) with high precision (1 kDa) (Liebthal et al., 2021; Wu et al., 2022; Young et al., 2018).

This study serves a dual purpose. First, it provides a quantitative characterization of GNE assembly and inhibition. To this end, we systematically investigated GNE assembly equilibria in the presence or absence of UDP‐GlcNAc, determining monomer–monomer (M:M) and dimer–dimer (D:D) binding affinities (*K*_D_ values) as indicators of GNE complex stability. The effects of the inhibitors, C5, C13, and C15, on these equilibria were quantitatively assessed using MP, yielding half maximal inhibitory concentration (IC_50_) values. These values were then converted to *K*_i_ values under the assumption of mixed competitive and allosteric inhibition—suggested by experimental results—using a modified Cheng–Prusoff equation (Equation S4) that accounts for allosteric effects induced by orthosteric inhibitors.

To demonstrate the applicability of our approach, we conducted a representative Schild analysis for inhibitor C15, applying a modified Schild equation (Equation S5) that incorporates both allosteric and competitive interactions observed in the inhibited GNE system. The resulting Schild plot and function allowed visualization of inhibition dynamics and quantification of the allosteric effect induced by the antagonist (inhibitor). While Schild analysis is widely used to evaluate antagonist potency and binding mechanisms, especially in competitive systems, its assumptions may not fully capture complex, cooperative behaviors (Lane et al., 2014). To address this, we additionally employed the Operational Model of Allosterically Modulated Agonism (OMAM, Equation S6) to fit substrate response data in the presence of C15 (Jakubík et al., 2020). This model enabled us to extract mechanistic parameters such as efficacy (*τ*), the substrate (*K*_S_) and inhibitor (*K*_B_) affinity to GNE, and cooperativity factors (*α* and *β*), thereby offering a more detailed and quantitative description of the allosteric inhibition observed. Finally, computational docking analyses provided atomistic insights into inhibitor binding, further supporting the mechanistic conclusions derived from both Schild and OMAM analyses.

Second, this study establishes MP as a powerful method for investigating protein assembly inhibition. Its single‐molecule, label‐free format enabled us to quantify how small molecules modulate GNE oligomerization. By analyzing monomer–monomer and dimer–dimer binding affinities (*K*_D_ values) and Hill coefficients, we characterized both binding dynamics and allosteric effects. IC_50_ values from concentration‐dependent inhibition curves were translated into *K*_i_ values, and a modified Schild and OMAM analysis for inhibitor C15 illustrated the applicability of the approach to assembly‐based inhibition mechanisms.

## 2 RESULTS

We systematically investigated GNE assembly dynamics under controlled conditions using recombinant His_6_‐tagged GNE, which exhibited an observed molecular mass of 46.4 kDa. The protein was prepared following previously published protocols by Gorenflos López, Dornan, et al. (2023) and Gorenflos López, Schmieder, et al. (2023). All experiments were conducted at 20°C in dulbecco's phosphate‐buffered saline (DPBS) (pH 7.4), with samples maintained in solution. To ensure thermodynamic equilibrium, each sample was incubated for 30 min before measurements. The optimal concentration for MP data collection (50 nM and four‐fold application dilution), as described in the methods section, was determined in initial empirical tests, providing reliable count rates without signal oversaturation and was used throughout this study.

To verify that the monomer–dimer–tetramer equilibrium of GNE remains stable during the 60‐second MP acquisition following dilution to 50 nM immediately before measurement, we performed two robustness tests under four representative conditions: (1) GNE at 800 nM (standard concentration in substrate titration and inhibition experiments), (2) GNE at 50 μM (the highest concentration used; Figure S1), (3) 800 nM GNE with 100 μM UDP‐GlcNAc (standard mixture in inhibition studies), and (4) the same mixture with the addition of 31.25 μM C15 (full tetramer inhibition by the most potent inhibitor; Figure S2). Across all conditions, the monomer–dimer–tetramer distribution remained unchanged during the 60‐second acquisition period after dilution to 50 nM. After 3 min post‐dilution, however, all samples showed a gradual shift from dimer toward monomer (6%–20%), while the tetramer fraction was unaffected in most cases, except in condition (3), where a modest 4%–7% increase was observed (Figure S2F).

### 2.1 GNE assembly equilibrium in the absence of UDP‐GlcNAc

**FIGURE 2:**
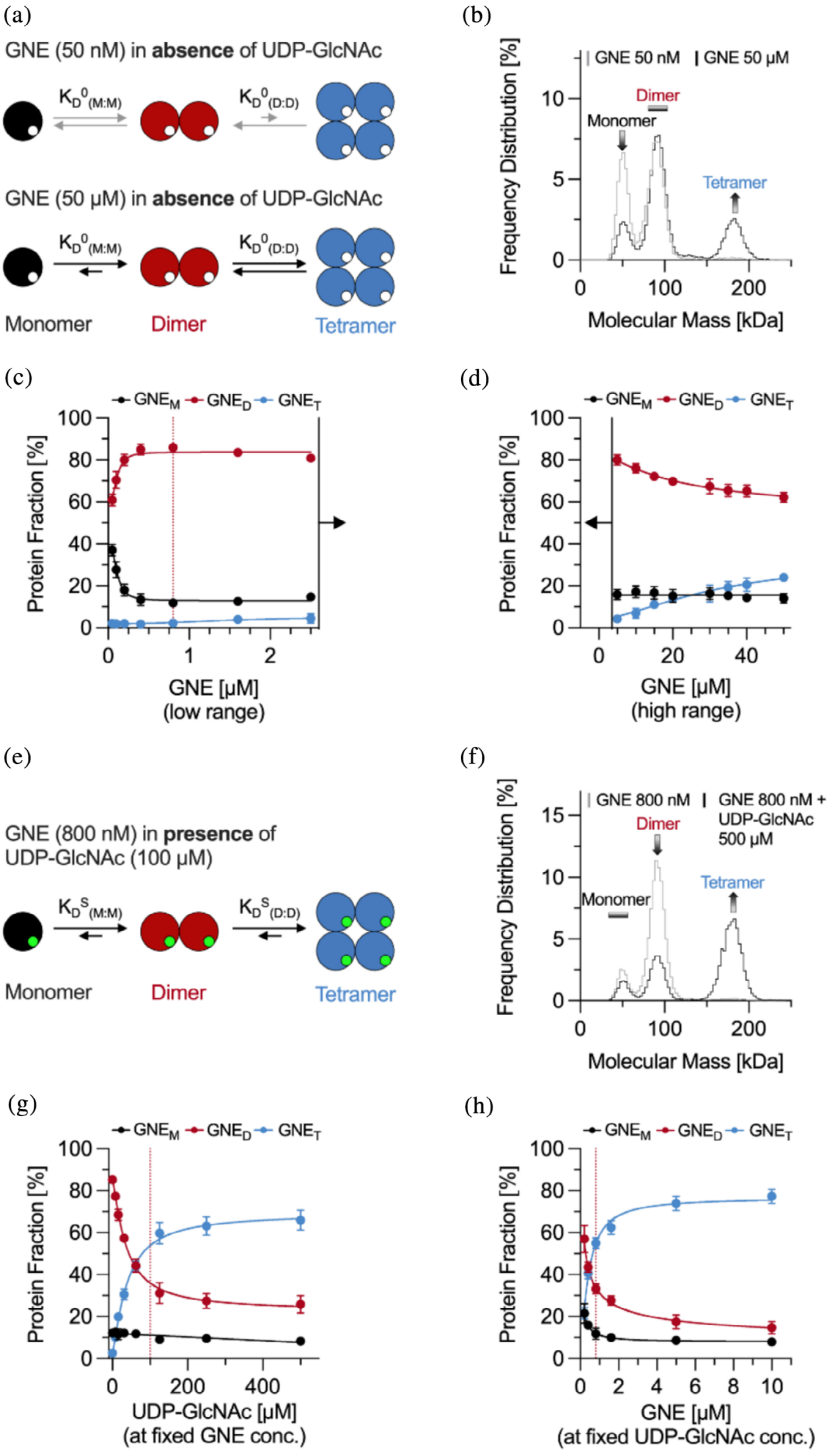
Assessment of uridine diphosphate‐*N*‐acetylglucosamine 2‐epimerase (GNE) assembly dynamics using mass photometry. (a) Schematic representation of monomer–dimer (M:D) and dimer–tetramer (D:T) equilibria in the absence of uridine diphosphate‐*N*‐acetylglucosamine (UDP‐GlcNAc) (0 indicates the absence of substrate), leading to affinity constants (*K*_D_^0^). Large circles represent GNE, while small empty circles denote unoccupied substrate‐binding sites. At a low GNE concentration (50 nM) the assembly equilibrium is shifted to monomers (gray arrows), while at a high GNE concentration (50 μM) the assembly equilibrium is shifted to tetramers (black arrows). (b) Merged mass photometry (MP) histograms of GNE at 50 μM (black, *N* = 20) and 50 nM (gray, *N* = 10) corresponding to the scheme in (a). (c and d) Effect of GNE concentration on its assembly distribution, expressed as protein fraction of each subunit (M = monomer, D = dimer, T = tetramer) relative to the total protein concentration, shown for low (c) and high (d) protein concentration regimes. The dotted red line marks the minimal GNE concentration (800 nM) required to reach a plateau in protein assembly, which was fixed for subsequent experiments. (e) Schematic representation of UDP‐GlcNAc‐dependent (S indicates the presence of substrate) GNE M:D and D:T equilibria (indicated by arrows), leading to affinity values (*K*_D_^S^). Green‐filled circles indicate UDP‐GlcNAc occupying the substrate‐binding pocket. (f) MP histograms of GNE (800 nM) in the absence (gray) and presence (black) of UDP‐GlcNAc (100 μM), corresponding to the scheme in (e). (g and h) Effect of UDP‐GlcNAc concentration on GNE assembly distribution. (g) Substrate titration against a fixed GNE (800 nM) and (h) GNE titration against a fixed substrate concentration (100 μM). In (g), the dotted red line indicates the minimal UDP‐GlcNAc concentration required to reach maximum tetramer formation. In (h), it marks the fixed GNE concentration (800 nM) used in subsequent experiments. All histograms include at least 8000 counts. The “Frequency Distribution” represents the percentage of detected counts corresponding to a specific mass relative to the total number of detected counts. Error bars indicate the standard deviation from at least three independent experiments, each measured in triplicate (*N* ≥ 9). Graphs are color‐coded to indicate GNE monomers (M, black), dimers (D, red), and tetramers (T, blue). Data fitting was carried out using a Hill‐based logistic model (Equation S1), adapted from the Kukura lab (Fineberg et al., 2020). For the tetramer curves shown in panels (g) and (h), the Hill coefficient was fixed at *n* = 1.3. For all other fits, *n* was left as a free parameter and served as an additional degree of freedom. *N* denotes the number of spectra or measurements combined to generate each merged spectra or data point.

To quantify GNE assembly equilibria in the absence of substrate, we performed MP experiments on a serial dilution of GNE in DPBS, with protein concentrations ranging from 50 nM to 50 μM (Figure 2a). Throughout the experiment, we identified three distinct molecular assemblies at ~50 kDa, ~90 kDa, and ~180 kDa, corresponding to monomers, dimers, and tetramers, respectively (Figures 2b and S3A,B).

Comparing these measured masses to the apparent monomer mass of 46.4 kDa, previously reported in our studies (Gorenflos López, Dornan, et al., 2023; Gorenflos López, Schmieder, et al., 2023), the MP results demonstrate 93% mass accuracy for the monomer and 97% for the dimer and tetramer using our experimental setup and mass standard. Between measurements, mass deviations remained within 1–5 kDa, indicating high reproducibility. As GNE concentration increased, we observed a shift in the protein assembly ratio toward higher molecular mass populations from 18:31:1 (M:D:T) at 50 nM to 4:18:7 at 50 μM (Figure 2a,b). Up to a GNE concentration of 800 nM, the dimer fraction increased, while monomer fractions decreased, reaching a stable multimer distribution over an extended concentration range. However, at protein concentrations above 5 μM, we observed a concomitant rise in tetramer formation and a decrease in dimer levels (Figure 2c,d). To determine protein affinities (*K*_D_) between monomers and dimers, protein fraction data ([GNE_M/D/T_]/[GNE_total_]) was plotted against the concentration of free monomer (GNE_M,free_) or free dimer (GNE_D,free_) (Figure S4A,B). Plateaus were constrained to the approximate plateau values determined in Figure 2c,d. The data were fitted using a Hill‐based logistic model (Equation S1) (Fineberg et al., 2020), derived from the law of mass action, with the Hill coefficient (*n*) used as a further degree of freedom, to fully describe the system. This analysis enabled us to determine the binding affinity constants (*K*_D_), which represent the half‐maximal conversion of monomer into dimer (*K*_D_^0^_(M:M)_) or dimer into tetramer (*K*_D_^0^_(D:D)_). We determined a monomer–monomer (M:M) interaction *K*_D_^0^_(M:M)_ of 12.6 ± 1.3 nM, while the dimer–dimer (D:D) interaction exhibited a *K*_D_^0^_(D:D)_ of 9.6 ± 2.2 μM. We also calculated affinity constants (*K*_D_^0^_Calc._) for monomer, dimer, or tetramer concentrations derived from MP ratios, applying the law of mass action. For monomer–monomer interactions, we obtained a mean *K*_D_^0^_Calc.(M:M)_ of 25.8 ± 3.7 nM, and for the dimer–dimer interactions, a mean *K*_D_^0^_Calc.(D:D)_ of 76.9 ± 10.1 μM. The calculated values are approximately two‐fold (M:M) and eight‐fold (D:D) higher than the corresponding affinities determined from the binding plots (Figure S4A,B). These values indicate that monomers associate more strongly to form dimers compared to dimers assembling into tetramers, highlighting a greater stability of the dimeric state in the absence of substrate.

### 2.2 Substrate effect on dimer and tetramer stability of GNE

To examine the impact of UDP‐GlcNAc (substrate) on GNE subunit affinities, we conducted a series of titration experiments (Figure 2e). In the first part of the experiment, UDP‐GlcNAc was titrated against a fixed GNE concentration (800 nM), which represents the minimum concentration required to establish a stable assembly equilibrium based on our previous dilution studies (Figure 2c). As the UDP‐GlcNAc concentration increased, we observed a progressive shift toward tetramer formation, which is clearly reflected in the MP spectra (Figures 2f and S3C,D). A plateau in tetramer formation was reached at 100 μM UDP‐GlcNAc, which was subsequently used as the standard concentration for all following experiments (Figure 2g). From the slope of the fitted curves (Equation S1) (Fineberg et al., 2020), we determined a Hill coefficient of *n* = 1.3 describing the tetramer fraction in the presence of the substrate, indicating moderate positive cooperativity. This value was fixed for fitting the tetramer data across subsequent inhibition experiments. We further determined, from the tetramer curve, a moderate substrate affinity (*K*_S_) of 34.6 ± 3.0 μM using Equation S2, which is close to the *K*_M_ (33.1 ± 4.2 μM) of GNE (Chen et al., 2016).

In the second part of the experiment, we performed the reverse titration, in which GNE was titrated against a fixed concentration of UDP‐GlcNAc (100 μM) (Figure 2h). Similar to the first titration, an increasing GNE concentration led to a continuous shift toward tetramer assembly.

To quantify the intermolecular affinities (*K*_D_) between monomers and dimers, we plotted the protein fraction data of the monomer and the tetramer against the concentration of free monomer (GNE_M,free_) or free dimer (GNE_D,free_) (Figure S4C,D) respectively and fitted the data using a Hill‐based logistic model (Equation S1) (Fineberg et al., 2020). The plateau values were constrained to the approximate asymptotes determined in Figure 2h. The fitted curves yielded *K*_D_ values of 37.2 ± 7.5 nM for M:M interactions and 98.1 ± 9.1 nM for D:D interactions, indicating that UDP‐GlcNAc promotes GNE oligomerization by significantly enhancing the stability of tetramers. For the measurements performed in the presence of substrate, mean *K*_D_^S^_Calc._ values were likewise determined using the law of mass action. A mean *K*_D_^S^_Calc.(M:M)_ of 65.3 ± 12.4 nM was obtained for the monomer–monomer interaction, and a mean *K*_D_^S^_Calc.(D:D)_ of 221.3 ± 27.2 nM for the dimer–dimer interaction. In both cases, the calculated affinity values (*K*_D_^S^_Calc._) were approximately two‐fold higher than the corresponding values derived from the fits (Figure S4C,D).

### 2.3 GNE assembly inhibition studies

Building on the stoichiometric distribution of GNE assemblies, as defined by the *K*_D_ values for dimers and tetramers under specific conditions, we further investigated the potency of three non‐carbohydrate‐based inhibitors (C5, C13, and C15) in destabilizing GNE dimers and tetramers, determining their respective IC_50_ values. To assess inhibition, we performed titration experiments, in which varying concentrations of inhibitors were tested against a constant GNE concentration (800 nM), both in the presence or absence of UDP‐GlcNAc (100 μM).

**FIGURE 3:**
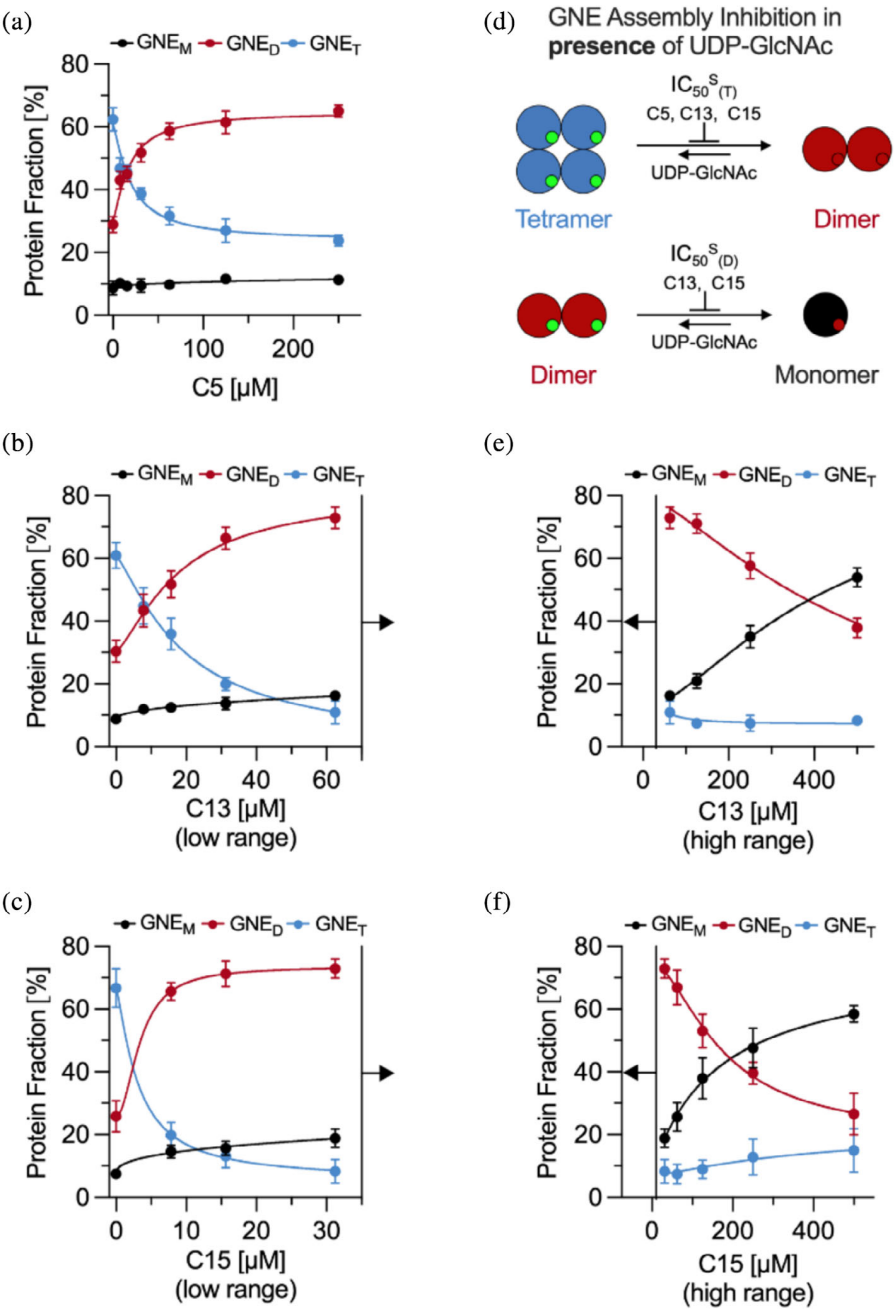
Inhibition of uridine diphosphate‐*N*‐acetylglucosamine 2‐epimerase (GNE) assembly by C5, C13, and C15 using mass photometry. The concentration‐dependent effects of inhibitors on the GNE (800 nM) tetramer and dimer stability were assessed using mass photometry. The monomer (M), dimer (D) or tetramer (T) fraction, expressed as a percentage of the total protein concentration, is shown for (a) C5, (b) C13 and (c) C15 at low inhibitor concentrations. (d) Schematic representation of the inhibition model, illustrating the equilibrium shift from tetramer (T, blue) to dimer (D, red) and dimer to monomer (M, black) upon inhibitor binding. This equilibrium was analyzed in the presence of 100 μM uridine diphosphate‐*N*‐acetylglucosamine (UDP‐GlcNAc), denoted by superscript S, to determine inhibitor‐specific IC_50_ values for tetramer (IC_50_^S^_(T)_) and dimer (IC_50_^S^_(D)_) destabilization. The large colored circles represent GNE protein assemblies, while small green and red filled circles indicate the substrate‐binding pocket occupied by UDP‐GlcNAc (green) or inhibitor (red). The inhibitor concentration‐dependent effects on GNE assembly distribution were also assessed at higher concentrations for (e) C13 and (f) C15 extending the lower concentration range shown in (b) and (c). Error bars represent the standard deviation from at least three independent experiments, each performed in technical triplicate (*N* ≥ 9). Data were fitted using a Hill‐based logistic model (Equation S3). For the tetramer curves, the Hill coefficient was fixed at *n* = 1.3, as determined in Figure 2g. For all other curves, *n* was left variable and treated as an additional degree of freedom. *N* indicates the number of measurements contributing to each data point.

In the presence of UDP‐GlcNAc, all inhibitors effectively destabilized tetramers (Figure 3a–c), as further illustrated schematically in Figure 3d and by the exemplary spectra (Figure S5). However, C13 and C15 also exhibited an additional destabilizing effect on dimers (Figure 3e,f). Notably, inhibition by C13 and C15 followed a sequential destabilization pattern, initially targeting tetramers, followed by dimers, allowing for the differentiation between low (Figure 3b,c) and high (Figure 3e,f) inhibitor concentration regimes (Figure S6).

In the absence of UDP‐GlcNAc, C13 and C15 significantly disrupted the dimeric assembly, whereas C5 had a minimal effect (Figure S7). By plotting protein fraction data against the inhibitor concentration, we determined IC_50_ values (Tables 1 and S1) using a Hill‐based logistic model (Equation S3). We further translated the IC_50_ values of the tetramer inhibition into assay‐independent *K*_i_ values, under the assumption of a mixed competitive and allosteric inhibition, using a Cheng–Prusoff equation (Equation S4), modified for allosteric assembly inhibition induced by orthosteric inhibitors. The IC_50_ and *K*_i_ values in the presence of substrate revealed an increasing inhibitory potency trend from C5 to C13 and C15. In the absence of the substrate (Figure S7), only C15 yielded an exact IC_50_ value.

**TABLE 1:** Quantitative analysis of inhibitor potency and binding energies for uridine diphosphate‐*N*‐acetylglucosamine 2‐epimerase (GNE) assembly disruption. The table summarizes experimentally and computationally derived parameters for the inhibition of GNE tetramers (T) by small molecules C5, C13, and C15. IC_50_ and *K*_i_ were determined by mass photometry in the presence of substrate (IC_50_^S^_(T)_, *K*_i_^S^_(T)_), using 100 μM uridine diphosphate *N*‐acetylglucosamine. Binding energy values (Δ*G*_Average,bind_, Δ*G*_bind_) were obtained from molecular docking studies and reflect the predicted interactions between each inhibitor and the GNE binding site. Superscripts S denote conditions with substrate. Errors represent the standard deviation from at least three independent experiments, each measured in triplicate.

	IC_50_^S^_(T)_ (μM)	*K*_i_^S^_(T)_ (nM)	Δ*G*_Average,bind_ ^a^ (kcal/mol)	Δ*G*_bind_ ^b^ (kcal/mol)	Freq.^c^
C5	19.7 ± 5.7	517.2 ± 210.4	−5.8	−7.6	17%
C13	19.4 ± 4.2	508.3 ± 182.0	−6.8	−7.0	67%
C15	3.1 ± 1.2	82.6 ± 39.7	−6.1	−6.6	48%

^a^ Δ*G*_Average,bind_ represents the average binding free energy (kcal/mol) calculated for a cluster of docking solutions within a 2 Å RMSD tolerance from the optimal binding pose.

^b^ Δ*G*_bind_ refers to the binding free energy (kcal/mol) of the most favorable docking solution, representing the lowest‐energy conformation identified in the docking studies.

^c^ Frequency (Freq.) indicates the percentage of docking simulations in which a given binding pose appears, serving as a measure of confidence in the predicted binding mode.

**FIGURE 4:**
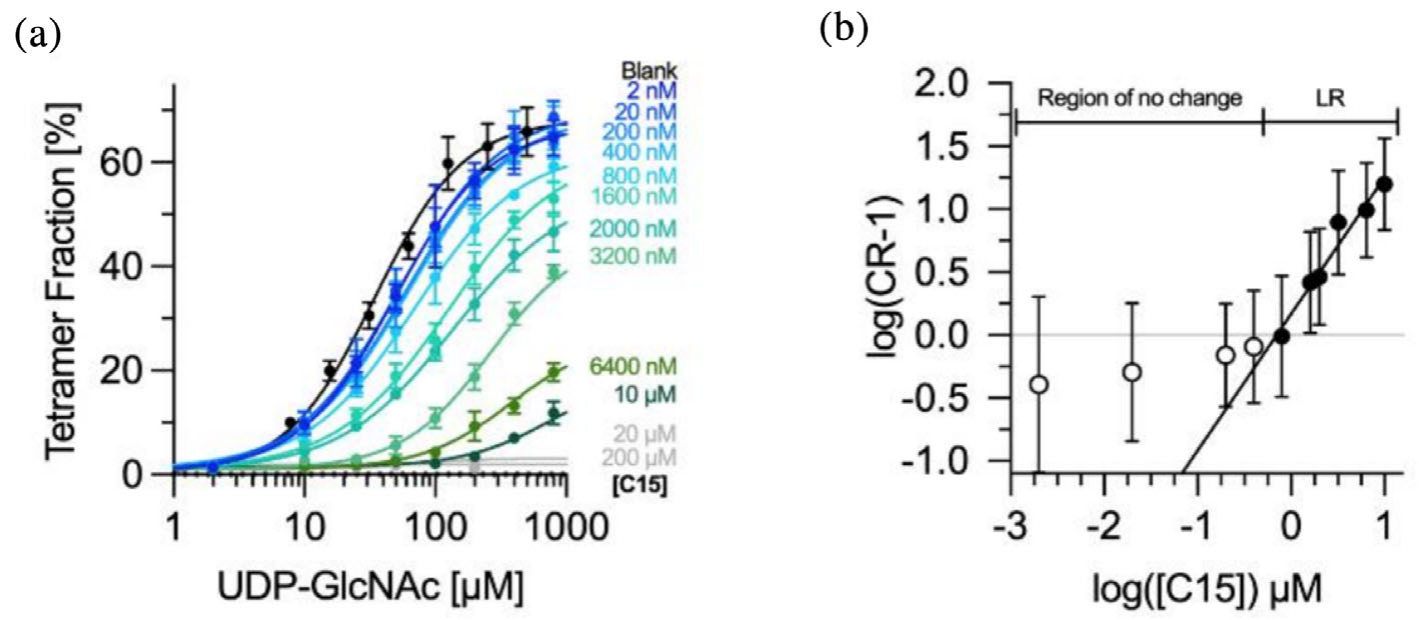
Schild analysis and Schild plot for uridine diphosphate‐*N*‐acetylglucosamine 2‐epimerase (GNE) tetramer inhibition by C15. (a) Mass photometry analysis of GNE tetramer assembly as a function of substrate concentration (uridine diphosphate‐*N*‐acetylglucosamine [UDP‐GlcNAc]) in the presence of increasing concentrations of inhibitor C15 (indicated by blue to green curves). Curves with no change are colored in gray. Tetramer fractions are shown as percentages of the total protein concentration. Data were fitted using Equation (S3) with a Hill coefficient *n* treated as an additional degree of freedom. (b) Double‐logarithmic Schild plot illustrating the inhibition of GNE tetramer assembly by C15. CR‐1 values were calculated as the ratio of EC_50_ values in the presence versus absence of inhibitor, minus one, based on the substrate titration curves in panel (a). Data points (filled circles) were fitted using a linear Schild equation (Equation S5). The plot is divided into two regions, indicated at the top: A low concentration “region of no change” and a linear region (LR) at higher C15 concentrations. The *x*‐intercept corresponds to the apparent log(*K*_B,app_). All measurements were performed at a fixed GNE concentration of 800 nM. Error bars represent the standard deviation (a), *N*(Blank) ≥ 9, *N*(C15:2 nM‐10 μM) = 6) or the standard error of the mean (SEM, b), *N*(C15:2 nM–10 μM) = 6) from at least two independent experiments, each measured in triplicate. *N* indicates the number of measurements included in each data point.

In addition to determining IC_50_ values, we sought to adapt the principles of Schild analysis to estimate the inhibitor affinity (*K*_B_) for GNE and to gain deeper insight into the underlying inhibitory mechanism. As a representative case, we focused on the most potent inhibitor, C15. For this purpose, we performed substrate titrations at a fixed GNE concentration (800 nM) in the absence or presence of increasing C15 concentrations. The resulting dose–response curves (Figure 4a), fitted using Equation (S3) (assuming EC_50_ = IC_50_) with a variable Hill coefficient, showed a progressive rightward shift in EC_50_ with increasing inhibitor concentration. This shift was accompanied by a reduction in maximal response amplitude, resulting in decreasing analysis accuracy at higher C15 concentrations, as visualized in Figure S8A. To quantify the shifts, EC_50_ values obtained in the presence of C15 (EC_50,Inh_) were normalized to the control (EC_50_ in absence of inhibitor), and transformed into concentration ratios (CR), from which CR‐1 values were plotted against inhibitor concentration in a double‐logarithmic Schild plot (Figure 4b). The resulting plot revealed two distinct regions: a no‐effect region at low concentrations (2 nM–0.4 μM) and a linear region (LR, 0.8–10 μM). The LR was analyzed using a standard linear Schild equation (Equation S5). The observed slope (*m* = 1.1) deviated from the classical value of 1 expected for purely competitive inhibition, indicating the presence of moderate cooperative effects. Due to this deviation, the large error bars causing an fit *R* ^2^ = 0.2, and the complexity of the inhibition profile, a precise dissociation constant for the inhibitor (*K*_B_) could not be obtained. Instead, an apparent affinity (*K*_B,app_ ≈ 0.7 μM) was estimated from the *x*‐intercept of the linear fit (Figure 4b).

To complement this analysis and gain mechanistic insight beyond the linear range, we applied the Hill‐modified OMAM (Equation S6) (Jakubík et al., 2020) to globally fit the full substrate‐response curves (Figure S8B). Key parameters, including maximal response (*E*_max_ = 68.3%), substrate affinity (*K*_S_ = 34.6 μM), were fixed based on prior analysis (Figure 2g). From the OMAM fit (Figure S8B), we extracted operational efficacy values: *τ*_S_ = 17.6 for the substrate and *τ*_I_ = 0.02 for the inhibitor, reflecting the strong tetramer‐inducing capacity of UDP‐GlcNAc and the negligible intrinsic efficacy of C15. Additionally, the model yielded a binding cooperativity factor (*ɑ* = 0.02), indicating strong negative cooperativity between C15 and substrate binding, consistent with allosteric modulation of the protein. The operational cooperativity factor (*β*  ≈ 0) suggests that C15 nearly abolishes the functional effect of the substrate upon binding, likely by inducing conformational changes that disrupt tetramer formation. This is consistent with the pronounced reduction in maximal response observed in the titration curves (Figure 4a). Notably, the inhibitor affinity (*K*_B_ = 25.2 nM) estimated from the OMAM global fit differs substantially from the apparent *K*_B,app_ obtained via Schild analysis of the LR.

### 2.4 Molecular modeling studies

**FIGURE 5:**
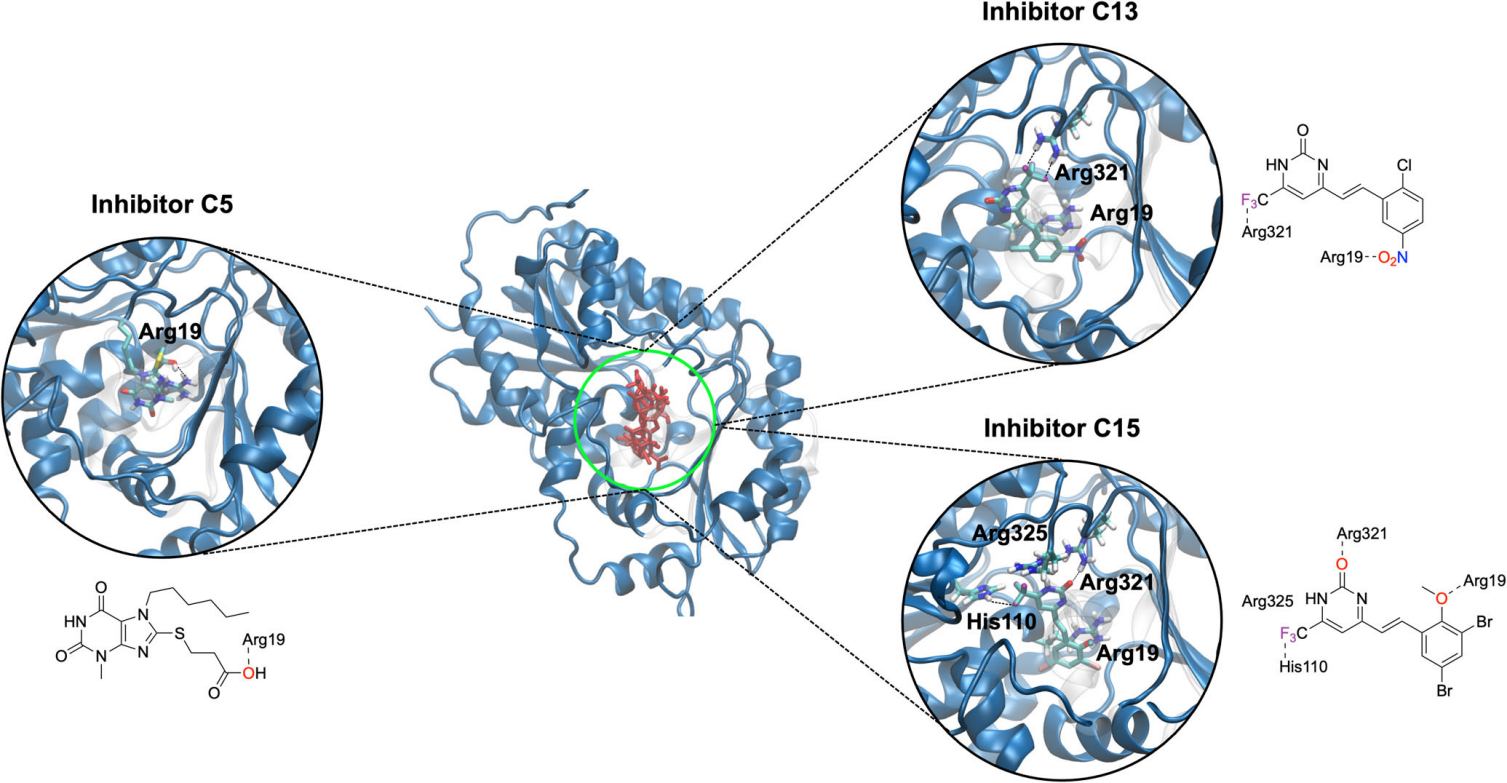
Docking analysis of inhibitor binding to the uridine diphosphate‐*N*‐acetylglucosamine 2‐epimerase (GNE) monomer. The central panel displays the GNE crystal structure (PDB, ID: 4zht) (Chen et al., 2016), with an overlay of the lowest‐energy, most populated docking poses (red) for all inhibitors. Independent docking results for C5 (left), C13 (top right), and C15 (bottom right) illustrate their respective binding orientation within the enzyme. Key interacting residues (depicted in licorice representation) within the GNE binding pocket are highlighted. The docking results reveal that all inhibitors occupy the substrate‐binding cleft, overlapping with the uridine diphosphate‐*N*‐acetylglucosamine binding site (green circle). The 2D structures of the inhibitors indicate chemical groups involved in interactions with GNE residues and were created using ChemDraw 22.2.0.3348 (PerkinElmer).

To atomistically explain the experimentally observed differences in inhibitor potency among C5, C13, and C15, and to gain deeper insights into their modes of action, we performed docking studies of the inhibitors with GNE monomers. Consistent with C5, both C13 and C15 exhibited preferential binding within the same protein pocket as UDP‐GlcNAc (Figure 5), supporting the inhibition mechanism proposed in our previous study based on HDX‐MS analysis and structural similarities between the inhibitors and the substrate (Gorenflos López, Dornan, et al., 2023). For each inhibitor, we identified the lowest‐energy binding pose and calculated the average binding energy (Δ*G*_Average,bind (T)_) for a cluster of poses within a 2 Å root mean square deviation (RMSD) tolerance from the optimal docking solution. The resulting binding energy values (Δ*G*_Average,bind_, ΔG_bind_), along with their frequency (Freq.) as a confidence metric, are summarized in Table 1. The energy values were obtained with considerable sampling frequencies, representing the percentage of simulation steps in which the inhibitor adopted a given binding pose. These results indicate an increasing inhibitor potency trend from C5 to C15 and C13, deviating slightly from our experimental findings.

To further interpret the docking results and gain deeper insight into the inhibition mechanism, we analyzed the key molecular interactions between the inhibitors and residues in GNE's substrate‐binding pocket (Figure 5). These interactions primarily differ in the number and positioning of hydrogen bonds (H‐bonds) formed between enzyme residues and inhibitor molecules, as illustrated in Figure 5. The carboxylic oxygen atom of C5 forms an H‐bond with Arg19, a key residue in the binding site. Similarly, C13 and C15 engage in H‐bonding at Arg19, with the oxygen of the C13 nitro group and the ether group of C15 interacting at this site. Additionally, the trifluoromethyl fluorine atoms of C13 and C15 establish H‐bonds with Arg321 and His110, respectively. C15 also forms an H‐bond between its 2‐pyrimidinone oxygen and Arg321, while the positive charge of Arg325 further stabilizes the interaction.

Notably, the H‐bond interactions at Arg19 and Arg321, which are common to all three inhibitors, are also observed in UDP binding to the GNE active site (Figure 1a). This suggests a competitive inhibition mechanism, where the inhibitors mimic substrate interactions, thereby blocking UDP‐GlcNAc binding and disrupting GNE function.

## 3 DISCUSSION

The enzymatic activity of GNE is inherently linked to its assembly state, with dimerization and tetramerization playing a crucial role in regulating its function (Gorenflos López, Dornan, et al., 2023). Due to the dynamic nature of GNE assembly, mass photometry (MP) provides an ideal tool for studying its assembly equilibria and the influence of inhibitors. In this study, we leveraged MP to comprehensively investigate GNE intermolecular stability in the presence or absence of UDP‐GlcNAc and to characterize the potency and mechanism of action of three non‐carbohydrate‐based inhibitors (C5, C13, and C15), previously identified in our research (Gorenflos López, Dornan, et al., 2023). Computational docking studies further supported these findings by providing atomistic insights into inhibitor binding affinities and potency trends.

Time‐resolved experiments under key conditions confirmed that the monomer–dimer–tetramer equilibrium remains unchanged during the 60‐second data acquisition immediately after a one‐step dilution to 50 nM. These results demonstrate that MP is well suited to study concentration‐dependent effects on the monomer–dimer–tetramer equilibrium across a broad concentration range.

Initial GNE titration experiments using MP revealed three distinct molecular assemblies corresponding to monomers (~50 kDa), dimers (~90 kDa), and tetramers (~180 kDa). The absence of trimeric intermediates suggests a stepwise assembly mechanism, transitioning from monomers to dimers and subsequently to tetramers, consistent with previous reports (Chen et al., 2016; Gorenflos López, Dornan, et al., 2023). In the absence of UDP‐GlcNAc, GNE predominantly existed as dimers, with strong monomer–monomer (M:M) affinity in the nanomolar range (*K*_D_^0^_(M:M)_ = 12.6 ± 1.3 nM) and weaker dimer–dimer (D:D) interactions in the micromolar range (*K*_D_^0^_(D:D)_ = 9.6 ± 2.2 μM). Substrate addition markedly shifted the equilibrium toward tetramer formation, increasing D:D affinity by two orders of magnitude and promoting strong positive cooperativity, as reflected for the tetramer by a Hill coefficient >1. Affinity values calculated using the law of mass action, based on protein concentrations determined from MP, were weaker and less precise than those obtained by graphical fitting with Equation S1. Nevertheless, all values remained within the same general range. This discrepancy likely arises because the mass‐action model does not account for the observed cooperative effects. Quantitative analysis of substrate titration data at a fixed GNE concentration (800 nM) further confirmed moderate substrate‐binding affinity (*K*_S_ = 34.6 ± 3.0 μM), and cooperativity, corroborating earlier HDX‐MS findings (Gorenflos López, Dornan, et al., 2023).

To investigate the inhibitory mechanisms of C5, C13, and C15, we previously assessed their effects on GNE dimer and tetramer stability at a single high inhibitor concentration in the presence of UDP‐GlcNAc. These semi‐quantitative comparisons suggested a potency ranking of C5 < C13 < C15, consistent with aSEC data and enzymatic activity assays (Gorenflos López, Dornan, et al., 2023). Subsequent inhibitor titration experiments allowed us to derive IC_50_ values (Tables 1 and S1), confirming this trend. Assuming a mixed competitive and allosteric inhibition mechanism, suggested by HDX‐MS data (Gorenflos López, Dornan, et al., 2023), molecular docking, and the observed Hill coefficient of the tetramer curves, we used a modified Cheng–Prusoff equation (Equation S4) to convert IC_50_ values into *K*_i_ values.

To gain further mechanistic insight, we modeled the concentration‐dependent inhibition data for the most potent inhibitor, C15. Modified Schild analysis revealed two regimes: no effect at low concentrations, and a linear range with slope *m* > 1 suggesting cooperativity. The Schild fit is expected to reach saturation in a third, non‐linear regime at C15 concentrations above 10 μM. Verification of this regime is experimentally not possible, as tetramer count rates become too low for reliable analysis, leading to disappearing amplitudes as shown in the reliability gradient. From the linear regime, we determined an apparent inhibitor affinity (*K*_B,app_) of ~0.7 μM. However, to more accurately capture the cooperative and allosteric behavior observed across the full dose–response range, we applied the OMAM (Equation S6) to globally fit the substrate‐response curves (Jakubík et al., 2020).

This model yielded efficacy (*τ*) and cooperativity (*α*, *β*) parameters: C15 showed minimal intrinsic efficacy (*τ*_I_ = 0.02) and strong negative binding cooperativity (*α* = 0.02), with almost complete suppression of substrate efficacy (*β* ≈ 0), consistent with the observed amplitude reduction in the titration curves. The inhibitor affinity derived from the OMAM fit (*K*_B_ = 25.2 nM) was ~28‐fold lower than the *K*_B,app_ obtained from the linear Schild analysis. This discrepancy underscores not only the limitations of Schild analysis in systems with cooperative interactions but also the fact that both affinity values are model‐based estimates. While OMAM provides a mechanistic and quantitative interpretation of such inhibition, definitive determination of inhibitor affinity would require direct structural or biophysical approaches, such as nuclear magnetic resonance spectroscopy titration or isothermal titration calorimetry (ITC).

Molecular docking supported a competitive binding mechanism, with all inhibitors occupying the UDP‐GlcNAc binding site and forming key interactions with the residues Arg19 and Arg321—both critical for substrate recognition. The number of hydrogen bonds formed correlated with the inhibitor potency, with C5, C13, and C15 forming one, two, and three stabilizing interactions, respectively, consistent with experimental trends.

In summary, this study provides a quantitative and mechanistic understanding of GNE assembly dynamics and its inhibition by non‐carbohydrate‐based inhibitors (C5, C13, and C15). By confirming the competitive nature of inhibition and identifying a cooperative component influencing GNE assembly stability, we expand upon the findings of our previous work (Gorenflos López, Dornan, et al., 2023). This work not only advances the mechanistic understanding of GNE regulation but also highlights the utility of MP and OMAM modeling for probing dynamic protein assembly and inhibitor mechanisms at the single‐molecule level.

## 4 MATERIALS AND METHODS

### 4.1 GNE expression and purification

The expression and purification of human UDP‐GlcNAc 2‐epimerase (GNE) were carried out following the protocol established by Chen et al. (2016). The GNE protein used in this work was expressed as a GNE with an *N*‐terminal His_6_‐tag in *Escherichia coli* BL21 (DE3) cells. Details on protein production and purification have been previously described in Gorenflos López, Dornan, et al. (2023).

### 4.2 UDP‐GlcNAc, C5, C13, C15, and GNE sample preparation

GNE sample aliquots were stored frozen at −80°C in Tris–HCl buffer (50 mM Tris–HCl, pH 8.0, 100 mM NaCl, 5% [v/v] glycerol, and 0.2 mM TCEP). Prior to use, samples were centrifuged (10 min, 30,000 × g, 4°C), and the protein concentration was determined using a NanoDrop2000c (Thermo Scientific, Cat# ND‐200C) at 280 nm. For concentration determination, the experimentally determined molar extinction coefficient (*ε* = 19,535 M/cm) and the molecular mass of the His_6_‐tagged monomer (46.4 kDa) were used (Gorenflos López, Dornan, et al., 2023; Gorenflos López, Schmieder, et al., 2023). Uridine diphosphate *N*‐acetyglucosamine disodium salt (UDP‐GlcNAc, Calbiochem, Cat# 670107) was prepared as a 10 mM stock solution in DPBS (−|−) buffer (Gibco, Cat# 14190144). Inhibitors 3‐((7‐hexyl‐3‐methyl‐2,6‐dioxo‐2,3,6,7‐tetrahydro‐1*H*‐purin‐8‐yl)thio)propanoic acid (C5, AKos GmbH, Cat# AKOS005519318), (*E*)‐4‐(2‐chloro‐5‐nitrostyryl)‐6‐(trifluoromethyl)pyrimidin‐2(1*H*)‐one (C13, AKos GmbH, Cat# AKOS024356555), and (*E*)‐4‐(3,5‐dibromo‐2‐methoxystyryl)‐6‐(trifluoromethyl)pyrimidin‐2(1*H*)‐one (C15, AKos GmbH, Cat# AKOS000638975) were dissolved in DMSO (Sigma‐Aldrich, Cat# 472301) as 10 mM stock solutions. All stock solutions were stored at −20°C until use.

### 4.3 Mass photometry experiments

All MP measurements were conducted using a OneMP (Refeyn) instrument equipped with a 525 nm green laser. To minimize background noise, the instrument was placed on an active vibration isolation system (Accurion i4 Series, Park Systems). High‐precision glass coverslips (Paul Marienfeld GmbH & Co. KG, Cat# 0107222) were thoroughly cleaned before use to ensure optimal measurement conditions. Prior to MP measurements, samples were incubated in phosphate buffer (DPBS[−|−], Gibco, Cat# 14190144) and low‐binding tubes for 30 min in a ThermoMixer C (Eppendorf, Cat# 5382000015) at 20°C to allow binding equilibrium to be reached. Immediately before measurement, a 50 nM working solution was prepared. Then, 5 μL of the working solution was added to a 15 μL DPBS (−|−) droplet on the glass coverslip, followed by mixing upon pipetting to ensure proper distribution before measurements began immediately. This approach provided high reproducibility in our results. For the measurements, a regular field of view was used. Data acquisition was performed over a 60‐s detection period with manual focus adjustment. Data were recorded using the software AquireMP (2024 R2, Refeyn) and referenced against a glycinin mass standard in DPBS (−|−). The glycinin standard, derived from defatted soybean flour, forms characteristic trimers (160 kDa), hexamers (320 kDa), nonamers (480 kDa), and dodecamers (640 kDa). Purified and lyophilized glycinin was kindly provided by the group of Dr. Rumiana Dimova (Chen et al., 2020; Mangiarotti et al., 2025). Data analysis and graphical visualization were performed using DiscoverMP (2024 R2, Refeyn) and GraphPad Prism 10 (10.1.1, Dotmatics). All experiments were conducted in at least three independent replicates, each performed as technical triplicates.

### 4.4 Affinity measurements of GNE subunits in the presence or absence of UDP‐GlcNAc

To determine the intermolecular protein affinities of GNE subunits, their assembly distribution was assessed using MP experiments. In the absence of substrate, serial GNE dilutions (50 μM to 50 nM) were performed to evaluate GNE subunit interactions. In the presence of UDP‐GlcNAc, intermolecular affinity changes were assessed through two titration approaches: First, different concentrations of UDP‐GlcNAc (500–7.81 μM) were titrated against a fixed concentration of GNE (0.8 μM). Second, different concentrations of GNE (0.2–10 μM) were titrated against a fixed concentration of UDP‐GlcNAc (100 μM). K_D_ values were obtained from binding curves (Figure S4), fitted using a Hill‐based logistic model (Equation S1) (Fineberg et al., 2020). The following dissociation constants were determined: *K*_D_^0^_(M:M)_ and *K*_D_^0^_(D:D)_ in the absence of UDP‐GlcNAc, *K*_D_^S^_(D:D)_ and *K*_D_^S^_(M:M)_ in the presence of UDP‐GlcNAc. Plateaus were constrained based on the asymptotic behavior of the GNE oligomer fraction shown in Figure 2c,d,h.

### 4.5 GNE assembly inhibition experiments

The effects of inhibitors (C5, C13, and C15) on GNE assembly were evaluated using MP. Twofold serial dilutions of the inhibitors (500–7.81 μM) were titrated against a constant GNE concentration (800 nM), either in the presence or absence of UDP‐GlcNAc (100 μM). The GNE and substrate concentrations were selected based on titration experiments ensuring binding saturation and the establishment of an assembly equilibrium. Prior to measurements, samples were incubated for 30 min at 20°C to allow equilibrium to be reached. IC_50_ values were determined from protein fraction vs. inhibitor concentration plots and fitted using a Hill‐based logistic function (Equation S3). Subsequently, *K*_i_ values for GNE inhibitors were derived from a Cheng–Prusoff equation accounting for allostery (Equation S4), detailed in Supporting Information S1.

### 4.6 GNE‐inhibitor Schild experiments

To assess the binding affinity of inhibitors to GNE, modified Schild experiments were performed using MP. Serial dilutions of UDP‐GlcNAc (800 μM to 100 nM) were titrated against a constant GNE concentration (800 nM) in the presence of varying concentrations of inhibitor C15 (200 μM to 2 nM). Enzyme and substrate concentrations were chosen based on prior titration experiments (Figure 2c,g) to ensure a stable oligomeric equilibrium. Inhibitor concentrations were selected around the IC_50_ to enable accurate response characterization. Samples were incubated for 30 min at 20°C to ensure equilibration before measurement. EC_50_ values were obtained from substrate titration curves (tetramer fraction vs. UDP‐GlcNAc concentration), fitted using a Hill‐based function (Equation S3). The resulting EC_50_ values in the presence of inhibitor (EC_50,Inh_) were converted into logarithmic concentration ratios (log(CR‐1)) and plotted against the corresponding logarithmic inhibitor concentrations to generate a Schild plot. Data points were then fitted using a linear Schild equation (Equation S5), as described in the Supporting Information S1. Further cooperative factors were obtained from fitting the substrate concentration‐dependent response curves globally with an OMAM model (Equation S6) (Jakubík et al., 2020).

### 4.7 Robustness test for GNE monomer–dimer–tetramer equilibrium stability

To confirm the signal stability of equilibrated GNE samples after dilution to 50 nM during the 60‐s data acquisition, we conducted two robustness tests. Following the sample preparation described in Section 4 (30 min equilibration at 20°C in DPBS (−|–)), four representative conditions were tested: (1) GNE at 800 nM (standard concentration in substrate titration and inhibition experiments), (2) GNE at 50 μM (highest concentration used), (3) GNE at 800 nM with 100 μM UDP‐GlcNAc (standard mixture in inhibition studies), and (4) GNE at 800 nM with 100 μM UDP‐GlcNAc and 31.25 μM C15 (full tetramer inhibition by the strongest inhibitor). Samples were measured using the standard procedure (dilution to 50 nM, application by mixing into a buffer droplet). In robustness test 1, 60‐s recordings were divided into three 20‐s intervals (0–20, 20–40, and 40–60 s), analyzed side by side, and compared to spectra from the standard 60‐s acquisition. In robustness test 2, the 50 nM dilutions were incubated for 3 or 5 min prior to data acquisition (60 s) and compared to spectra obtained immediately after dilution. In both tests, data were evaluated as merged spectra and visualized in time‐resolved bar plots.

### 4.8 Docking studies

Molecular docking studies were performed using Autodock 4.2.33 (Morris et al., 2009), by using the crystal structure of GNE (PDB ID: 4zht) (Chen et al., 2016). Ligand coordinates were generated using Avogadro 1.2.0 (Hanwell et al., 2012). Both proteins and ligands were preprocessed with the AutoDock Tools 1.5.7 (ADT) package to merge nonpolar hydrogens, calculate Gasteiger charges, and select the rotatable side‐chain bonds (Morris et al., 2009; Sanner, 1999). For docking evaluation, grid boxes were set with a spacing of 0.375 Å and dimensions of 60 × 60 × 60 points, centered on a binding pocket identified using the MDpocket program (Schmidtke et al., 2011). Grid maps were subsequently generated using AutoGrid 4.2, which is included in the AutoDock 4.2 distribution (Morris et al., 2009). All docking simulations were conducted using default AutoDock parameters (Morris et al., 2009).

## AUTHOR CONTRIBUTIONS

**Nico Boback:** Investigation; writing – original draft; methodology; visualization; writing – review and editing; formal analysis; data curation. **Jacob Gorenflos López:** Resources; writing – review and editing. **Christian P. R. Hackenberger:** Funding acquisition; writing – review and editing. **Santiago Di Lella:** Investigation; funding acquisition; writing – original draft; writing – review and editing; visualization; methodology; formal analysis. **Daniel C. Lauster:** Conceptualization; funding acquisition; writing – original draft; methodology; writing – review and editing; visualization; project administration; supervision; formal analysis; resources.

## CONFLICT OF INTEREST STATEMENT

The authors declare no competing interests.

## Supporting information


**Data S1.** Supporting Information.

## Data Availability

The data that support the findings of this study are available from the corresponding author upon reasonable request.
