# Supplementary material for: Quantitative analysis of inhibitor‐induced assembly disruption in human UDP‐GlcNAc 2‐epimerase using mass photometry
Source: Protein Sci. 2025 Oct 16;34(11):e70335. doi: 10.1002/pro.70335 (PMC12529881; doi:10.1002/pro.70335)
Supplement: Supplementary file 1 — Data S1. Supporting Information. [file PRO-34-e70335-s001.docx]

**Quantitative analysis of inhibitor-induced assembly disruption in human UDP-GlcNAc 2-epimerase using mass photometry**

Nico Boback,^1^ Jacob Gorenflos López,^2,3^ Christian P. R. Hackenberger,^2,3^ Santiago Di Lella,^4^ and

Daniel C. Lauster^1,5,^*

^1^Freie Universität Berlin, Institute of Pharmacy, Biopharmaceuticals, Kelchstr. 31, 12169 Berlin, Germany

^2^Leibniz-Forschungsinstitut für Molekulare Pharmakologie (FMP), Robert-Rössle-Str. 10, 13125 Berlin, Germany

^3^Humboldt-Universität zu Berlin, Department of Chemistry, Brook-Taylor-Str. 2, 12489 Berlin, Germany

^4^Instituto de Química Biológica de la Facultad de Ciencias Exactas y Naturales, Universidad de Buenos Aires –

Consejo Nacional de Investigaciones Científicas y Técnicas, C1428EHA, Ciudad de Buenos Aires, Argentina

^5^Lead contact

*Correspondence: daniel.lauster@fu-berlin.de

# Table of Content

[Table of Content 1](#_Toc205729530)

[Supporting Information 3](#_Toc205729531)

[Equation S1. Hill-based logistic fitting function for determination of dissociation constants (K_D_). 3](#_Toc205729532)

[Equation S2. Hill function for the determination of substrate affinity to the enzyme and maximal response in substrate titration experiments in absence of inhibitor. 3](#_Toc205729533)

[Equation S3. Hill-based fitting function for IC_50_ determination. 3](#_Toc205729534)

[Equation S4. Modified Cheng-Prusoff equation for the IC_50_ conversion into K_i_ for allosteric protein assembly inhibition. 3](#_Toc205729535)

[Equation S5. Linear Schild fitting function. 4](#_Toc205729536)

[Equation S6. Hill modified operational model of allosterically modulated agonism (OMAM) for the modelling of Schild-like experiments. 4](#_Toc205729537)

[Figure S1. Test for signal stability of equilibrated GNE solutions during data acquisition 5](#_Toc205729538)

[Figure S2. Test for signal stability of equilibrated GNE solutions in the presence of substrate and inhibitor during data acquisition 6](#_Toc205729539)

[Figure S3. Merged non-normalized mass-photometry spectra of GNE in the absence or presence of UDP-GlcNAc shown in Figure 2 (main article). 7](#_Toc205729540)

[Figure S4. Mass photometry analysis of GNE fractions in binding plots. 8](#_Toc205729541)

[Figure S5. Merged non-normalized mass-photometry spectra of GNE in the presence of UDP-GlcNAc at key concentrations of C5, C13 and C15 shown in Figure 3 (main article). 9](#_Toc205729542)

[Figure S6. Concentration-dependent effects of C13 and C15 on GNE assembly at constant UDP-GlcNAc levels. 9](#_Toc205729543)

[Table S1. Inhibitory potency of C5, C13, and C15 against GNE dimers in the presence or absence of UDP-GlcNAc. 10](#_Toc205729544)

[Figure S7. Concentration-dependent GNE assembly inhibition in the absence of UDP-GlcNAc with increasing concentrations of inhibitors C5, C13 or C15. 10](#_Toc205729545)

[Figure S8. Quantitative analysis of cooperative inhibition of GNE tetramer formation by C15 and visualization of data reliability with rising C15 levels. 11](#_Toc205729546)

[Derivations 12](#_Toc205729547)

[Derivation S1. Hill-based inhibition function 12](#_Toc205729548)

[Derivation S2. Modification of the Cheng-Prusoff equation considering competitivity and cooperativity. 14](#_Toc205729549)

[Derivation S3. Determination of inhibitor affinities (K_B_) accounting for allostery using adapted Schild plot and function 15](#_Toc205729550)

[References 16](#_Toc205729551)

# Supporting Information

$$\text{f}\left( \text{[GNE]} \right)\text{ = }\text{M}_{\text{1}}\text{ + }\left( \text{M}_{\text{2}}\text{ - }\text{M}_{\text{1}} \right)\left( \frac{\text{[GNE]}^{\text{n}}}{\text{(}\text{[GNE]}^{\text{n}}\text{ + }{\text{K}_{\text{D}}}^{\text{n}}\text{)}} \right)$$

## Equation S1. Hill-based logistic fitting function for determination of dissociation constants (K_D_).

This equation describes the protein fraction f([GNE])) as a function of GNE protein concentration ([GNE]), where f([GNE]) represents the fraction of a specific GNE assembly state (X), normalized to the total GNE concentration ([GNE_X_]/[GNE_total_]), where X denotes monomer (M), dimer (D), or tetramer (T). M_1_ and M_2_ define the lower and upper plateau regions of the protein fraction. K_D_ represents the dissociation constant, quantifying the equilibrium between free and assembled protein. The Hill coefficient (n) represents an additional degree of freedom. For tetramer curves in presence of the substrate saturation, n = 1.3 determined in **Figure 2G** (main article) was fixed, indicating the degree of cooperativity in the assembly process. The equation is based on the law of mass action and was adapted from Fineberg *et al*. in a modified way.^1^

$$\text{f([S]) = }\frac{\text{E}_{\text{max}}\text{ ∙} \text{[S]}^{\text{n}}}{{\text{K}_{\text{S}}}^{\text{n}}\text{ ∙} \text{[S]}^{\text{n}}}$$

## Equation S2. Hill function for the determination of substrate affinity to the enzyme and maximal response in substrate titration experiments in absence of inhibitor.

This equation models the protein fraction f([S]) as a function of the substrate concentration [S]. E_max_ (68.3%) represents the maximal possible response (in our case maximal percentage of tetramer fraction under defined conditions), K_S_ the substrate affinity to the enzyme and n = 1.3 the Hill-coefficient, determined in **Figure 2G** (main article).

$$\text{f}\left( \left[ \text{I} \right] \right)\text{ }\text{=}\text{ }\text{M}_{\text{1}}\text{ }\text{+}\text{ }\text{(}\text{M}_{\text{2}} \text{-}\text{ }\text{M}_{\text{1}}\text{)}\frac{\text{1}}{\text{1}\text{ }\text{+}\text{ }\left( \frac{\text{I}\text{C}_{\text{50}}}{\left[ \text{I} \right]} \right)^{\text{n}}}$$

## Equation S3. Hill-based fitting function for IC_50_ determination.

The equation models the protein fraction f([I]) of individual protein subunits (M = monomer, D = dimer, T = tetramer) as a function of the inhibitor concentration ([I]). M_1_ and M_2_ define the minimal and maximal plateau regions of the fraction of assembled protein. IC_50_ represents the half-maximal inhibitory concentration and n the Hill-coefficient, treated as an additional degree of freedom. To fit tetramer fractions in presence of substrate saturation, a n = 1.3 was fixed, determined in **Figure 2G** (main article). This function is derived from the rate law of mass action, as detailed in **Derivation S1**.

$$\text{K}_{\text{i}}\text{ = }\left( \frac{\text{I}\text{C}_{\text{50}}}{\text{1 + }\left( \frac{\text{[S]}}{\text{K}_{\text{0.5}}} \right)^{\text{n}}} \right)\text{ ∙ }\text{ɑ}$$

## Equation S4. Modified Cheng-Prusoff equation for the IC_50_ conversion into K_i_ for allosteric protein assembly inhibition.

Modified equation for converting IC_50_ values into assay-independent inhibition constants (Kᵢ) for competitive and allosteric protein assembly inhibition. IC_50_ denotes the inhibitor concentration required for half-maximal inhibition, determined from titration experiments (**Table 1**). K_0.5_ represents the half-maximal assembly saturation (K_0.5,(D:D)_ = 9.6 µM), derived from GNE dilution assays. [S] is the substrate concentration used (100 µM), n is the Hill coefficient (n = 1.3), determined in **Figure 2G** (main article), and α is a correction factor (α_D_ = 0.3, α_T_ = 0.5) accounting for the oligomeric nature of the protein mixture. The derivation and modification of the equation are provided in **Derivation S2**.

$$\text{log(CR-1) = f([I]) = }\text{m log([I]) - m }\text{log(K}_{\text{B}}\text{)}$$

## Equation S5. Linear Schild fitting function.

This equation, adapted in modified form from Lane *et al.*,^2^ describes the linear region of the Schild plot. f([I]) represents the logarithmic form of the competitive concentration ratio parameter, log(CR-1), where CR is defined as the ratio of EC_50_ in the presence (EC_50,Inh_) to that in the absence of inhibitor. The function characterizes the effect of an inhibitor on protein assembly in the presence of the substrate (UDP-GlcNAc) across varying inhibitor concentrations ([I]). m denotes the slope of the linear fit (typically m > 1 in cooperative systems) and K_B_ is the apparent dissociation constant of the antagonist. A detailed derivation is provided in **Derivation S3**.

$$\text{f([S]) = E = }\frac{\text{E}_{\text{max}}\text{ ∙ (}\text{τ}_{\text{S}}\text{ ∙ }\text{[S]}^{\text{n}}\text{ }\text{∙}\text{ }\left( {\text{K}_{\text{B}}}^{\text{n}}\text{+ α ∙}\text{ }\text{β ∙}\left[ \text{I} \right]^{\text{n}} \right)\text{ }\text{+ }\text{τ}_{\text{I}}\text{ ∙}\left[ \text{I} \right]^{\text{n}}\text{ }\text{∙}\text{ }{\text{K}_{\text{S}}}^{\text{n}}\text{)}}{\left[ \text{S} \right]^{\text{n}}\text{ }\text{∙}\text{ }{\text{K}_{\text{B}}}^{\text{n}}\text{ }\text{+}\text{ }{\text{K}_{\text{S}}}^{\text{n}}\text{ }\text{∙}\text{ }{\text{K}_{\text{B}}}^{\text{n}}\text{ }\text{+}\text{ }\left[ \text{I} \right]^{\text{n}}\text{ }\text{∙}\text{ }{\text{K}_{\text{S}}}^{\text{n}}\text{ }\text{+ α∙}\text{ }\left[ \text{S} \right]^{\text{n}}\text{ }\text{∙}\text{ }\left[ \text{I} \right]^{\text{n}}\text{ }\text{+ }\text{τ}_{\text{S}}\text{ }\text{∙}\text{ }\left[ \text{S} \right]^{\text{n}}\text{ }\text{∙}\text{ }\left( {\text{K}_{\text{B}}}^{\text{n}}\text{ }\text{+ α}\text{ }\text{∙}\text{ }\text{β}\text{ }\text{∙}\text{ }\left[ \text{I} \right]^{\text{n}} \right)\text{ }\text{+}\text{ }\text{τ}_{\text{I}}\text{ }\text{∙}\text{ }\text{[I]}^{\text{n}}\text{ }\text{∙}\text{ }{\text{K}_{\text{S}}}^{\text{n}}}$$

## Equation S6. Hill modified operational model of allosterically modulated agonism (OMAM) for the modelling of Schild-like experiments.

This equation describes the response f([S]) = E as a function of the orthosteric substrate concentration ([S]) in presence of varying allosteric inhibitor concentrations ([I]). Cooperativity is described by the factors ɑ (binding cooperativity), β (operational cooperativity) and operational efficacies by 𝛕_S_ and 𝛕_I_ for the substrate and the inhibitor respectively. E_max_ denotes the maximal possible response (maximal tetramer fraction, 68.3%) determined in **Figure 2G** (main article). Dissociation constants are named K_S_ (34.6 µM) for the substrate, determined in **Figure 2G** (main article), using **Equation S2** and K_B_ for the inhibitor. n is the Hill-coefficient treated as an additional degree of freedom, introduced for a better fit of the concentration-response curves. The equation was adapted in modified form from Jakubík *et al.*.^3^


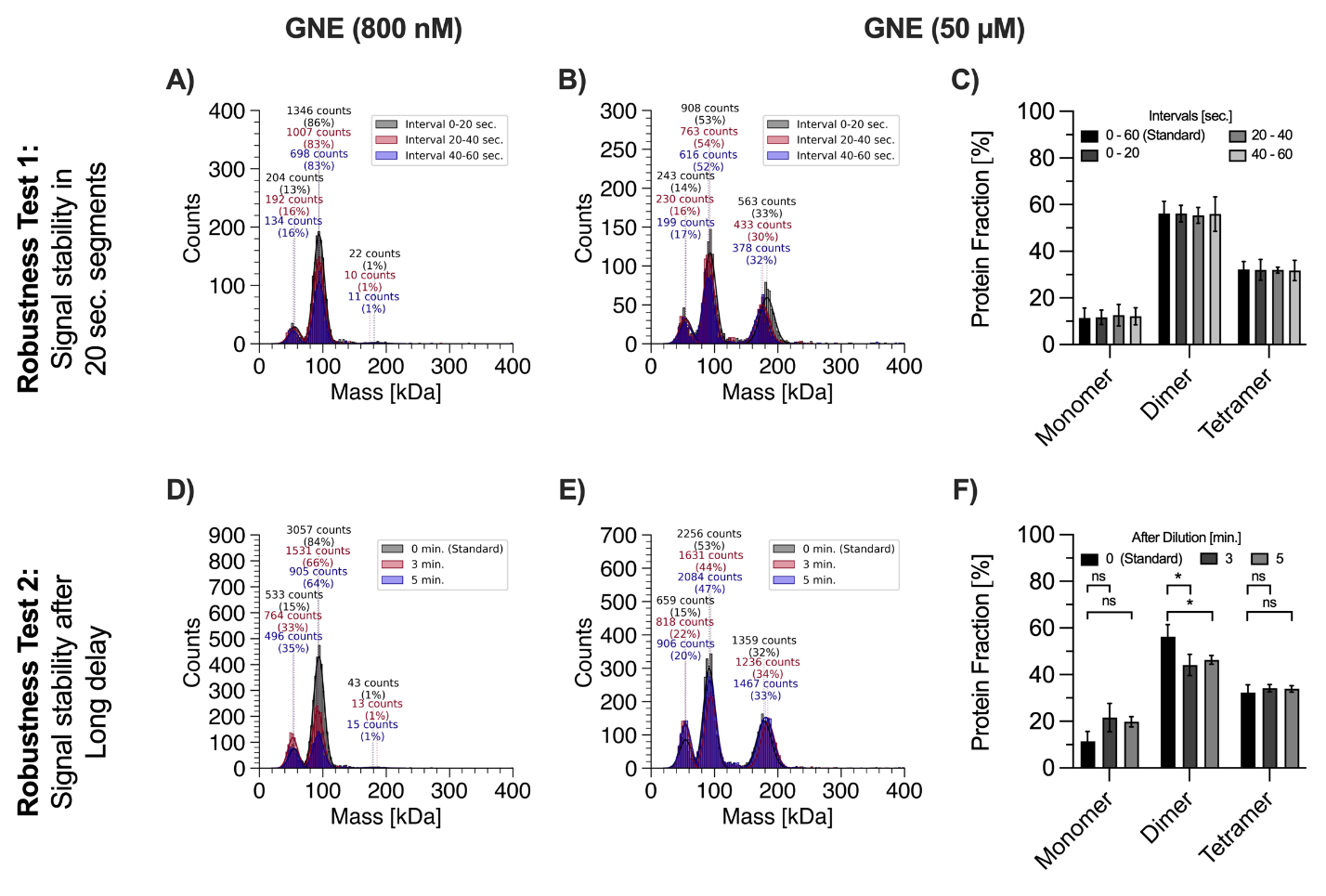


## Figure S1. Test for signal stability of equilibrated GNE solutions during data acquisition

In order to test the signal stability of data acquisition for GNE, two different robustness tests were performed. The sample preparation was done as outlined in the method section (30 minutes at 20°C in DPBS (-|-)) for 800 nM and 50 µM GNE concentrations. The time between sample equilibration and start of the data acquisition (deadtime) was 5 seconds (standard offset). A)-C) In robustness test 1, the 60 second data recording was split into three 20-seconds intervals (0-20, 20-40, 40-60 seconds). The spectra of each time interval are shown in A) for 800 nM and B) 50 µM GNE. D)-F) In robustness test 2, samples (D) 800 nM, E) 50 µM) were incubated up to 5 minutes before the sampling started. C), F) All obtained data are also represented exemplarily for 50 µM GNE in bar plots and statistically compared (One-way ANOVA, Dunnett’s multiple comparison test) to the standard procedure. Statistical significance is indicated as follows: ns, not significant (p > 0.1234); p < 0.1234 (*); p < 0.0332 (**), p < 0.0021 (***). The “Protein Fraction” denotes the proportion of the number of counts of each assembly state (monomer, dimer, tetramer) in the total number of counts in %. All merged spectra and error bars indicate the results of 3 independent replicates (N = 3).


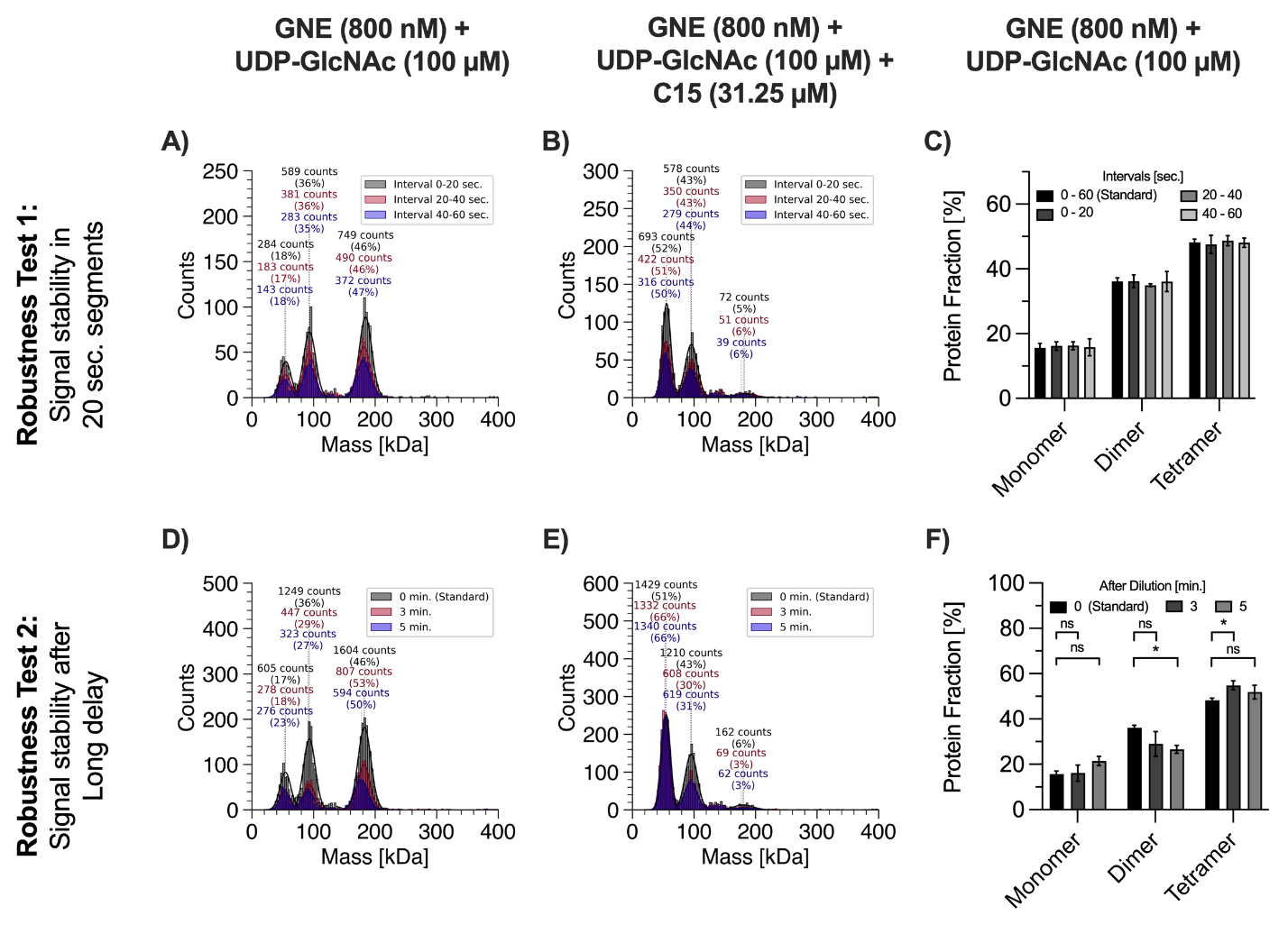


## Figure S2. Test for signal stability of equilibrated GNE solutions in the presence of substrate and inhibitor during data acquisition

In order to test the signal stability of data acquisition for GNE, two different robustness tests were performed. The sample preparation was done as outlined in the method section (30 minutes at 20°C in DPBS (-|-)) for 800 nM + 100 µM UDP-GlcNAc and 800 nM GNE + 100 µM UDP-GlcNAc + 31.25 µM C15 concentrations. The time between sample equilibration and start of the data acquisition (deadtime) was 5 seconds (standard offset). A)-C) In robustness test 1, the 60 second data recording was split into three 20-seconds intervals (0-20, 20-40, 40-60 seconds). The spectra of each time interval are shown in A) for 800 nM + 100 µM UDP-GlcNAc and B) 800 nM GNE + 100 µM UDP-GlcNAc + 31.25 µM C15. D)-F) In robustness test 2, samples (D) 800 nM + 100 µM UDP-GlcNAc, E) 800 nM GNE + 100 µM UDP-GlcNAc + 31.25 µM C15) were incubated up to 5 minutes before the sampling started. C), F) All obtained data are also represented exemplarily for 800 nM + 100 µM UDP-GlcNAc in bar plots and statistically compared (One-way ANOVA, Dunnett’s multiple comparison test) to the standard procedure. Statistical significance is indicated as follows: ns, not significant (p > 0.1234); p < 0.1234 (*); p < 0.0332 (**), p < 0.0021 (***). The “Protein Fraction” denotes the proportion of the number of counts of each assembly state (monomer, dimer, tetramer) in the total number of counts in %. All merged spectra and error bars indicate the results of 3 independent replicates (N = 3).


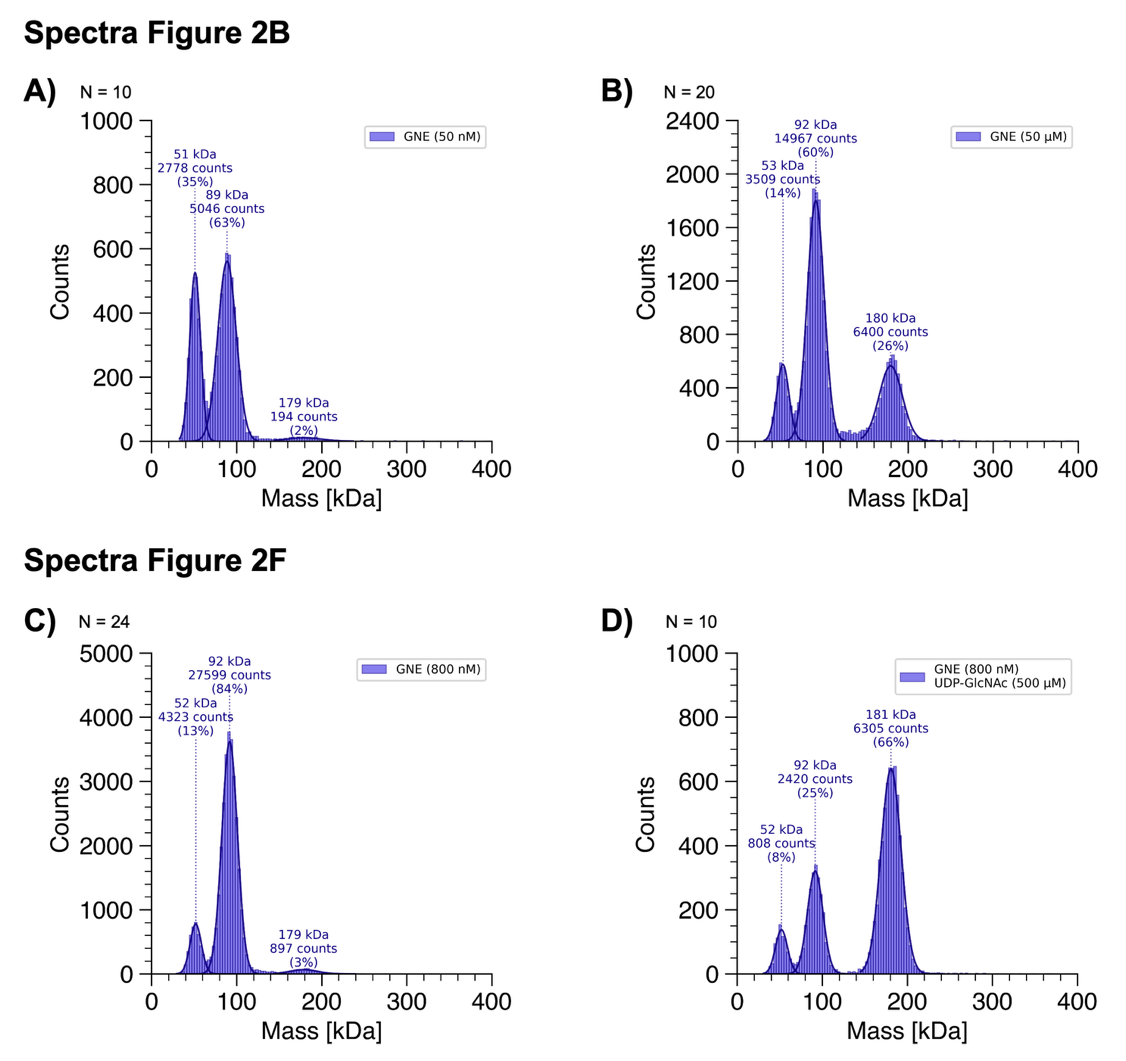


## Figure S3. Merged non-normalized mass-photometry spectra of GNE in the absence or presence of UDP-GlcNAc shown in Figure 2 (main article).

Merged mass photometry spectra of A) GNE (50 nM, N = 10), B) GNE (50 µM, N = 20), C) GNE (800 nM, N = 24), and D) GNE (800 nM) + UDP-GlcNAc (500 µM, N = 10). The x-axes show the molecular mass in kDa, whereas the y-axes give the number of particle-counts per 3 kDa bin. For every signal the label quotes the Gaussian-fit mean mass, the associated counts, and their fractional contribution to the total counts measured in %. N denotes the number of individual spectra merged to generate each spectrum.


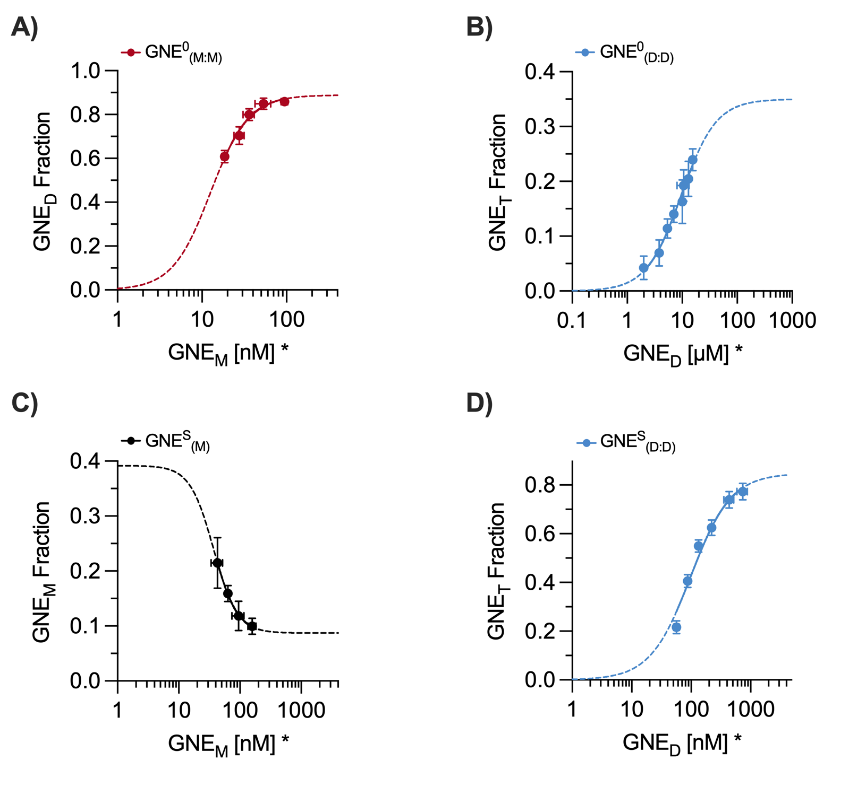


## Figure S4. Mass photometry analysis of GNE fractions in binding plots.

Binding curves illustrating the dissociation constants (K_D_) for GNE subunit interactions (monomer-monomer (M:M, red, black), dimer-dimer (D:D, blue)) in presence (S) or absence (0) of UDP-GlcNAc. A) Binding curve for monomer-monomer interactions in the absence of UDP-GlcNAc, yielding K_D_^0^_(M:M)_ for dimer formation. B) Binding curve for dimer-dimer interactions in the absence of UDP-GlcNAc, yielding K_D_^0^_(D:D)_ for tetramer formation. C) Binding curve for monomer-monomer interactions in the presence of UDP-GlcNAc (S), yielding K_D_^S^_(M:M)_. D) Binding curve for dimer-dimer interactions in the presence of UDP-GlcNAc (S), yielding K_D_^S^_(D:D)_. The GNE fraction represents the **proportion of monomer, dimer or tetramer populations** relative to the **total protein concentration** in the analyzed solution. **GNE_M,free_** and **GNE_D,free_** refer to the **concentrations of free monomers and dimers**, respectively. **Error bars** indicate the **standard deviation from at least three independent experiments, each measured in triplicate (N ≥ 9)**. Due to the **logarithmic scale, error bars in the x-direction may not always be visible. Binding curves were fitted using Equation S1, with the Hill coefficient treated as an additional degree of freedom except in panel D), where n = 1.3 (as determined in Figure 2G of the main article) was fixed. X-axes show calculated values (indicated by *).**


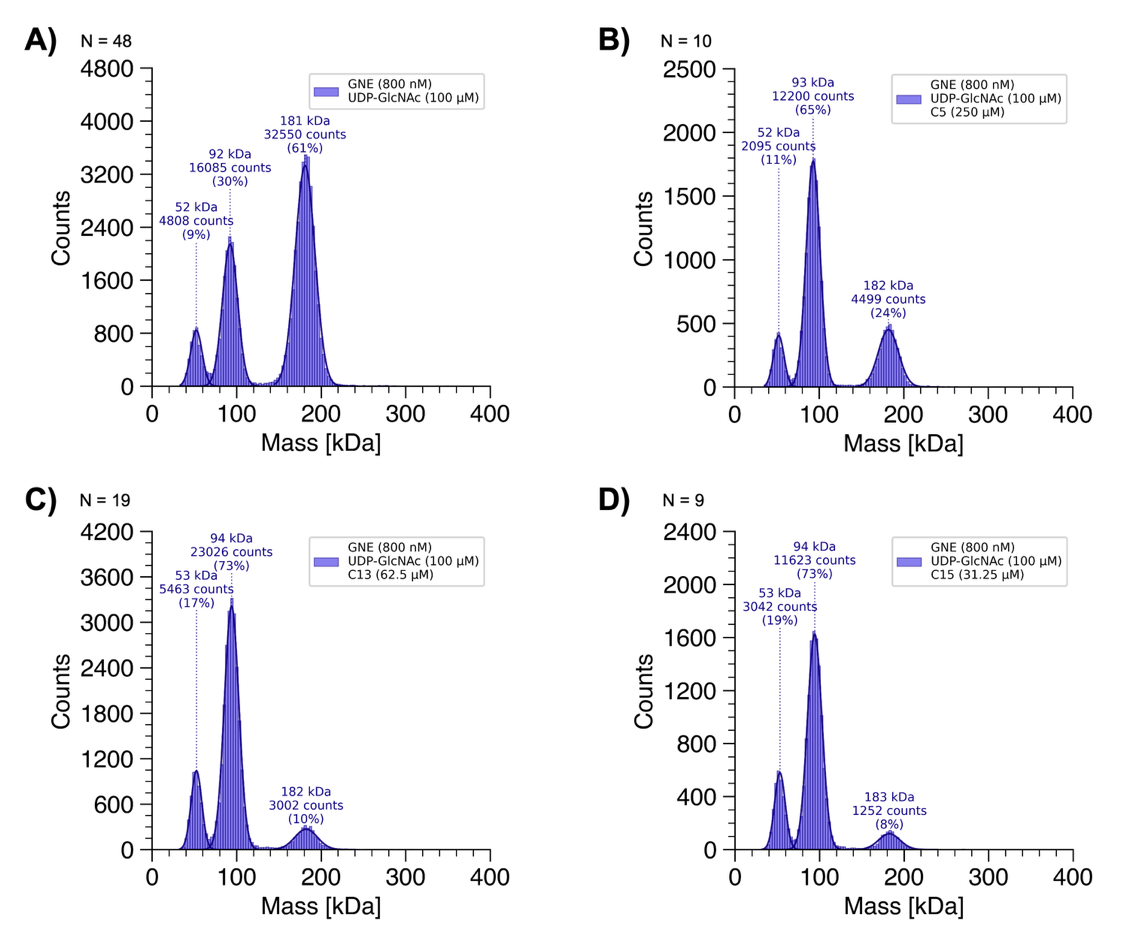


## Figure S5. Merged non-normalized mass-photometry spectra of GNE in the presence of UDP-GlcNAc at key concentrations of C5, C13 and C15 shown in Figure 3 (main article).

Merged mass photometry spectra of GNE (800 nM) + UDP-GlcNAc (100 µM) in presence of A) Blank (N = 48), B) C5 (250 µM, N = 10), C) C13 (62.5 µM, N = 19), and D) C15 (31.25 µM, N = 9). The x-axes show the molecular mass in kDa, whereas the y-axes give the number of particle-counts per 3 kDa bin. For every signal the label quotes the Gaussian-fit mean mass, the associated counts, and their fractional contribution to the total counts measured in %. N denotes the number of individual spectra merged to generate each spectrum.

**
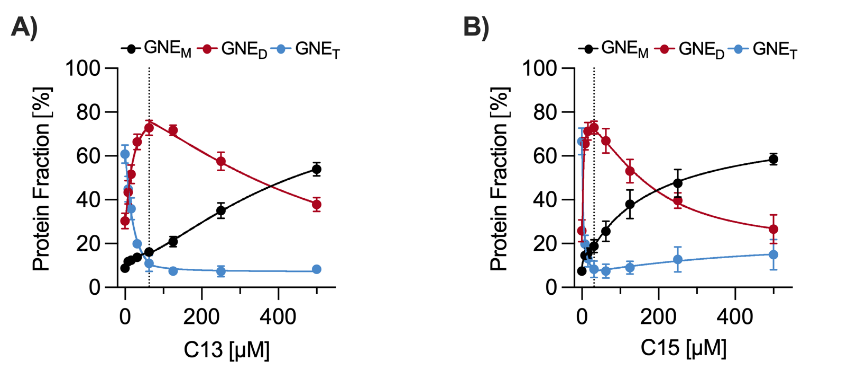
**

## Figure S6. Concentration-dependent effects of C13 and C15 on GNE assembly at constant UDP-GlcNAc levels.

Effect of inhibitor concentration on GNE assembly, shown as the protein fraction of each subunit: monomer (M, black), dimer (D, red), tetramer (T, blue) expressed as a percentage of the total protein concentration. Impact on GNE assembly is determined for A) C13, and B) C15. The dotted grey lines separate low and high concentration regimes, which were analyzed individually (see **Figure 3** in the main article). Each concentration regime was fitted independently using **Equation S3**, with a Hill-coefficient of n = 1.3 for the tetramer curves, determined in **Figure 2G** (main article). For the other curves n was left variable and treated as an additional degree of freedom. Error bars indicate the standard deviation from at least three independent experiments, each measured in triplicate (N ≥ 9).

## Table S1. Inhibitory potency of C5, C13, and C15 against GNE dimers in the presence or absence of UDP-GlcNAc.

Experimentally and computationally derived potency values (IC_50_) for GNE dimer (D) inhibition by C5, C13, and C15. Measurements were performed using mass photometry in the presence (IC_50_^S^_(D)_) or absence (IC_50_^0^_(D)_) of 100 µM UDP-GlcNAc. **S and 0** denote conditions **with and without substrate**, respectively. Reported errors represent the standard deviation from at least two independent experiments, each measured in triplicate (N^S^ ≥ 9, N^0^ = 6).

|  | IC_50_^S^_(D)_ [µM] | IC_50_^0^_(D)_ [µM] |
| --- | --- | --- |
| C5 | - | - |
| C13 | 471.9 ± 42.5 | >1000 |
| C15 | 172.7 ± 100.1 | 21.0 ± 2.6 |


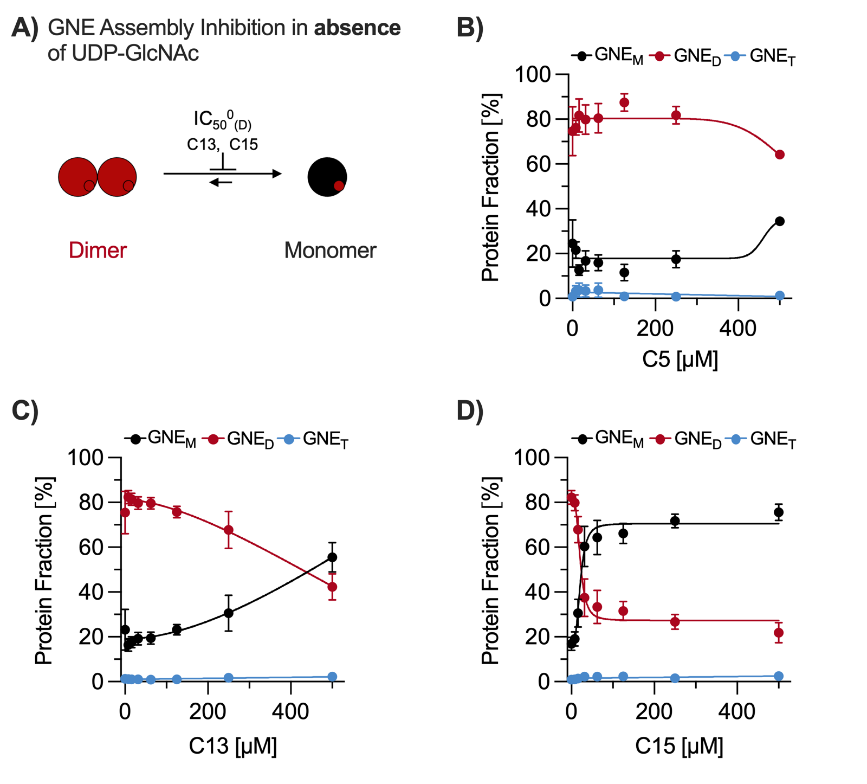


## Figure S7. Concentration-dependent GNE assembly inhibition in the absence of UDP-GlcNAc with increasing concentrations of inhibitors C5, C13 or C15.

A) Schematic representation of the inhibitor effect (small red spheres) on the monomer-dimer equilibrium in absence of UDP-GlcNAc. Arrows indicate a shift towards the monomer population upon inhibitor binding. IC_50_ values were determined from the following titration experiments. Concentration-dependent effects of inhibitors B) C5, C) C13, D) C15 on GNE subunit distribution, represented as the protein fraction of each subunit: monomer (M, black), dimer (D, red), and tetramer (T, blue) as a percentage of the total protein concentration. Error bars indicate the standard deviation from two independent experiments, each measured in triplicate (N = 6). Curves were fitted using **Equation S3**. The Hill coefficient (n) was left variable and treated as an additional degree of freedom. Data points of the tetramer populations, that were not possible to fit, due to a too low fractions, were connected *via* strait lines. N denotes the number of measurements included in each datapoint.


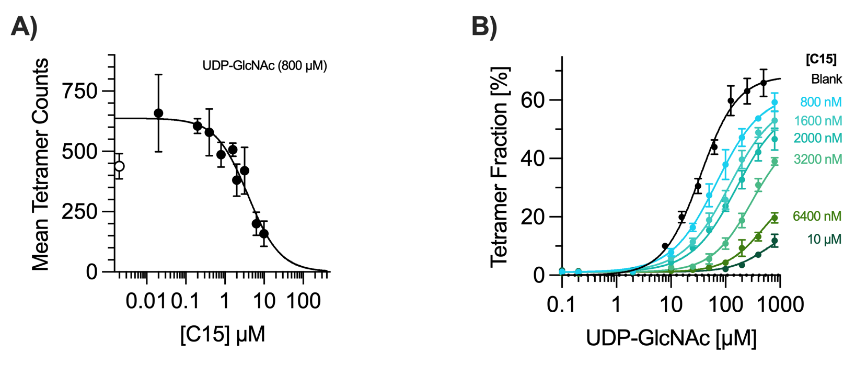


## Figure S8. Quantitative analysis of cooperative inhibition of GNE tetramer formation by C15 and visualization of data reliability with rising C15 levels.

A) Inhibitor concentration dependent number of tetramers counts in presence of 800 µM UDP-GlcNAc (last data point) showing the decreasing accuracy of Schild experiments at higher C15 concentrations. White filled dots represent data neglected in the fit. B) Substrate-dependent GNE tetramer formation analyzed by mass photometry and fitted using a Hill-modified Operational Model of Allosterically Modulated Agonism (OMAM, **Equation S6**) to extract efficacy (𝛕_S_, 𝛕_I_) and cooperative parameters (ɑ, β).^3^ A fixed maximal response (E_max_ = 68.3%), substrate affinity (K_A_ = 34.6 µM), determined in **Figure 2G** (main article), were used. Error bars represent the standard deviation of at least two independent experiments, each measured in triplicate (N_Blank_ ≥ 9, N_C15:2nM-10µM_ = 6).

# Derivations

## Derivation S1. Hill-based inhibition function

The inhibition of protein assembly (A) can be described by a simplified binding equilibrium. In the case of GNE, A denotes either the dimeric or tetrameric form of the enzyme. The inhibitor is represented by I, and m indicates the number of inhibitor molecules that bind to the assembly.

A + m I ⇋ AI_m_

Applying the law of mass action at thermodynamic equilibrium yields the following expression, where the ratio of the inhibitor-bound protein assembly ([AI_m_]) to the unbound components [A] and [I]) defines the dissociation constant:

$\text{K}_{\text{D}}\text{ = }\frac{\left[ \text{A} \right] \left[ \text{I} \right]^{\text{m}}}{\text{[A}\text{I}_{\text{m}}\text{]}}$ (1)

To account for cooperative binding, arising from multiple inhibitor binding sites within the assembly, the Hill coefficient (n) is introduced:

$\text{K}_{\text{D}}\text{ = }\frac{\left[ \text{A} \right] \left[ \text{I} \right]^{\text{n}}}{\text{[A}\text{I}_{\text{m}}\text{]}}$ (2)

Rearranging for the concentration of the inhibitor-bound assembly gives:

$\left[ \text{A}\text{I}_{\text{m}} \right]\text{ }\text{=}\text{ }\frac{\left[ \text{A} \right] \left[ \text{I} \right]^{\text{n}}}{\text{K}_{\text{D}}}$ (3)

Assuming that the total concentration of the assembly remains constant throughout the experiment, the following mass balance equation applies:

$\text{[A]}_{\text{total}}\text{ = [A]}$ (4)

Substituting equation (3) into (4):

$\left[ \text{A} \right]_{\text{total}}\text{ = }\left[ \text{A} \right]\text{ + }\frac{\left[ \text{A} \right] \left[ \text{I} \right]^{\text{n}}}{\text{K}_{\text{D}}}$ (5)

Factoring out [A]:

$\left[ \text{A} \right]_{\text{total}}\text{ }\text{=}\text{ }\left[ \text{A} \right] \left( \text{1}\text{ }\text{+}\text{ }\frac{\left[ \text{I} \right]^{\text{n}}}{\text{K}_{\text{D}}} \right)$ (6)

The fraction of inhibitor-bound protein assembly relative to the total is then given by:

$\frac{\text{[}\text{A}_{\text{m}}\text{]}}{\left[ \text{A} \right]_{\text{total}}}\text{ = }\frac{\frac{\left[ \text{I} \right]^{\text{n}}}{\text{K}_{\text{D}}}}{\text{1 + }\frac{\left[ \text{I} \right]^{\text{n}}}{\text{K}_{\text{D}}}}$ (7)

Simplifying:

$\frac{\text{[A}\text{I}_{\text{m}}\text{]}}{\left[ \text{A} \right]_{\text{total}}}\text{ }\text{=}\text{ }\frac{\left[ \text{I} \right]^{\text{n}}}{\text{K}_{\text{D}}\text{ }\text{+}\text{ }\left[ \text{I} \right]^{\text{n}}}$ (8)

To define the half-maximal inhibitory concentration (IC_50_), we introduce the condition, where 50 % of the protein assembly is inhibited. At this inhibitor concentration, [I] = IC_50_.

Considering cooperative binding, the relationship between IC_50_ and K_D_ is given by ${\text{IC}_{\text{50}}}^{\text{n}}$ = K_D_. Substituting K_D_ in equation (8) and dividing numerator and denominator by [I]^n^ yields the inhibitory form of the Hill equation:

$\frac{\text{[A}\text{I}_{\text{m}}\text{]}}{\left[ \text{A} \right]_{\text{total}}}\text{=}\frac{\text{1}}{\text{1}\text{ }\text{+}\text{ }\left( \frac{\text{I}\text{C}_{\text{50}}}{\left[ \text{I} \right]} \right)^{\text{n}}}$ (9)

To allow for sigmoidal fitting of experimental data, two scaling parameters, M_1_ and M_2_, are introduced to define the minimum and maximum plateaus of the inhibition curve, respectively. The resulting equation describes the fraction of inhibited protein assemblies as a function of inhibitor concentration, allowing for determination of IC_50_:

$\text{f}\left( \left[ \text{I} \right] \right)\text{ }\text{=}\text{ }\text{M}_{\text{1}}\text{ }\text{+}\text{ }\text{(}\text{M}_{\text{2}} \text{-}\text{ }\text{M}_{\text{1}}\text{)}\text{ }\frac{\text{1}}{\text{1}\text{ }\text{+}\text{ }\left( \frac{\text{I}\text{C}_{\text{50}}}{\left[ \text{I} \right]} \right)^{\text{n}}}$ (10)

## Derivation S2. Modification of the Cheng-Prusoff equation considering competitivity and cooperativity.

The Cheng-Prusoff equation is used to translate IC_50_ values, obtained in enzyme activity inhibition studies, into assay-independent, comparable K_i_ values. The regular Cheng-Prusoff equation considering competitive inhibition requires the half-maximal inhibitory concentration (IC_50_), the substrate concentration ([S]) and the Michaelis-Menten constant (K_M_). To apply on assembly inhibition, the K_M_ can be replaced by the affinity constant K_D_, derived from substrate titration experiments.

$\text{K}_{\text{i}}\text{ }\text{=}\text{ }\frac{\text{I}\text{C}_{\text{50}}}{\text{1}\text{ }\text{+}\text{ }\frac{\text{[S]}}{\text{K}_{\text{M}}}}\text{ }\text{=}\text{ }\frac{\text{I}\text{C}_{\text{50}}}{\text{1}\text{ }\text{+}\text{ }\frac{\text{[S]}}{\text{K}_{\text{D}}}}$ (1)

Cooperativity requires the implantation of the Hill-coefficient (n), since the binding behavior is not following the classic Michaelis-Menten model. Further an additional oligomeric correction factor (ɑ) has to be included, considering the ratio of aimed assembly (dimer, tetramer) to the total protein concentration. The affinity measure is included with the half-maximal substrate saturation (K_0.5_).

$\text{K}_{\text{i}}\text{ = }\frac{\text{I}\text{C}_{\text{50}}}{\left( \text{1 + }\frac{\text{[S]}}{\text{K}_{\text{0.5}}} \right)^{\text{n}}}\text{ }\text{∙}\text{ }\text{ɑ}$ (2)

## Derivation S3. Determination of inhibitor affinities (K_B_) accounting for allostery using adapted Schild plot and function

To quantify a mixed competitive and allosteric inhibition of GNE assembly in a manner, independent of substrate concentrations, we employed the Schild equation, which is traditionally used to determine antagonist affinity for receptors in presence of an agonist, to derive the binding constant of an antagonist (K_B_).

The Schild equation uses the concentration ratio parameter (CR-1) to quantify the competitive effect of the antagonist (in our case inhibitor) on the agonist (in our case substrate) potency. The **CR-1** parameter is defined as the ratio of the half-maximal effective agonist concentration in the **presence** (EC_50_,_modulated_) and **absence** (EC_50,control_) of the antagonist (in this case, the **inhibitors**) minus one:

$\text{CR - 1 = }\frac{\text{EC}_{\text{50,modulated}}}{\text{EC}_{\text{50,control}}}\text{ - 1}$ (1)

For our study, we determined EC_50_ values from **Protein Fractions (PF) *vs.* substrate concentration plots, derived from substrate titration experiments in presence or absence of the inhibitor.** The PF represent the proportion of monomers, dimers, and tetramers (derived from protein counts) as a percentage of the total protein concentration in solution. The **effectivity parameter** is defined as the ratio of the **EC_50,Inh_** in the presence of the inhibitor **(indicated by subscript Inh)** to the **EC_50_ in absence of the inhibitor, minus one**:

$\text{CR - 1 = }\frac{\text{EC}_{\text{50,Inh}}}{\text{EC}_{\text{50}}} \text{-}\text{ }\text{1}$ (2)

The resulting logarithmic values were plotted against the logarithmic inhibitor concentration (log([I])).

**Linear Schild Equation**

To fit the linear part of the Schild-plot, we adapted the Schild equation from literature describing **agonist-antagonist affinity interactions** at receptors.^2^

$\text{log(CR - 1) = log}\left( \text{[B]} \right) \text{- log(}\text{K}_{\text{B}}\text{)}$ (3)

Due to the additional cooperative effect, besides the competitive, the slope is deviating from 1, therefore we introduced the factor m, that describes the slope of the resulting curve. [B] is replaced by [I] for the inhibitor concentration.

$\text{log(CR-1) = f([I]) = }\text{m log([I]) - m }\text{log(K}_{\text{B}}\text{)}$ (4)

# References

1. Fineberg, A., Surrey, T., and Kukura, P. (2020). Quantifying the Monomer–Dimer Equilibrium of Tubulin with Mass Photometry. Journal of Molecular Biology *432*, 6168-6172. <https://doi.org/10.1016/j.jmb.2020.10.013>.

2. Lane, J.R., Donthamsetti, P., Shonberg, J., Draper-Joyce, C.J., Dentry, S., Michino, M., Shi, L., López, L., Scammells, P.J., Capuano, B., et al. (2014). A new mechanism of allostery in a G protein-coupled receptor dimer. Nat Chem Biol *10*, 745-752. 10.1038/nchembio.1593.

3. Jakubík, J., Randáková, A., Chetverikov, N., El-Fakahany, E.E., and Doležal, V. (2020). The operational model of allosteric modulation of pharmacological agonism. Scientific Reports *10*, 14421. 10.1038/s41598-020-71228-y.
